# Integrated transcriptomic and proteomic analysis of cancer-suppressive *Mesocestoides corti* larvae

**DOI:** 10.1186/s13071-026-07391-4

**Published:** 2026-04-26

**Authors:** Vojtěch Vajs, Libor Mikeš, Roman Leontovyč, Madina Tulpová, Lucie Korená, Ondřej Tolde, Tomáš Macháček, Jan Brábek, Daniel Rösel, Petr Horák

**Affiliations:** 1 Department of Parasitology, Faculty of Science, Charles University, Prague, Czech Republic; 2 Department of Cell Biology, Faculty of Science, Biotechnology and Biomedicine Centre of the Academy of Sciences and Charles University (BIOCEV), Vestec, Czech Republic

**Keywords:** Cancer, Tumor suppression, Tapeworm, *Mesocestoides*, Kunitz, Proteome, Transcriptome

## Abstract

**Background:**

The larvae of *Mesocestoides corti* have been previously shown to abrogate the growth and metastasis of the highly aggressive B16F10 melanoma in mice. In order to investigate the potential ways in which this effect is mediated, we present, to the best of our knowledge, the most comprehensive analyses of *M. corti* larval molecular data so far, while listing and exploring various potentially immunomodulatory molecules found therein. We show expression and protein abundance in larvae under in vivo and in vitro conditions. A promising ortholog, *M. corti* Kunitz-type chymotrypsin-specific inhibitor 1 (McKI-C1) of a known cancer-suppressive Kunitz protein from *Echinococcus granulosus* is also tested, both in vitro and in vivo.

**Methods:**

In order to explore the potential effector mechanisms and molecules behind its cancer-suppressive capabilities, we analyzed the *M. corti* larval transcriptome in the C57BL/6J and ICR mouse strains, as well as in vitro. The proteomic profiles of whole homogenate and excretory-secretory products of tetrathyridia were analyzed by nano-shotgun liquid chromatography with tandem mass spectrometry and thoroughly annotated. A Kunitz protein candidate (McKI-C1) was recombinantly expressed in *Escherichia coli*, biochemically characterized, and functionally tested for its anti-cancer effect with mouse melanoma B16F10 cells and mouse ovarian carcinoma ID8 cells.

**Results:**

We present, to the best of our knowledge, the most extensive list of experimentally verified *M. corti* protein products. Many proteins potentially responsible for this tapeworm’s immune-related cancer-suppressive abilities and immunomodulation were found within its proteome and transcriptome. These include numerous members of the superfamily of cysteine-rich secretory proteins, such as glioma pathogenesis-related-like proteins, and Kunitz-domain proteins. Functional tests of recombinant McKI-C1 performed both in vitro and in vivo did not confirm its expected tumor-suppressing properties. Therefore, the exact effectors of this tapeworm’s likely immune-mediated anti-tumor capabilities need to be examined in further studies.

**Conclusions:**

The transcriptomic and proteomic analysis of *M. corti* larvae carried out in the present study produced an extensive list of promising immunomodulatory or cancer-suppressive molecules. While the molecule chosen for analysis in the present study, McKI-C1, did not show any effect in vitro or in vivo, the immunomodulatory or cancer-suppressive potential of the other experimentally verified molecules remains of interest.

**Graphical Abstract:**

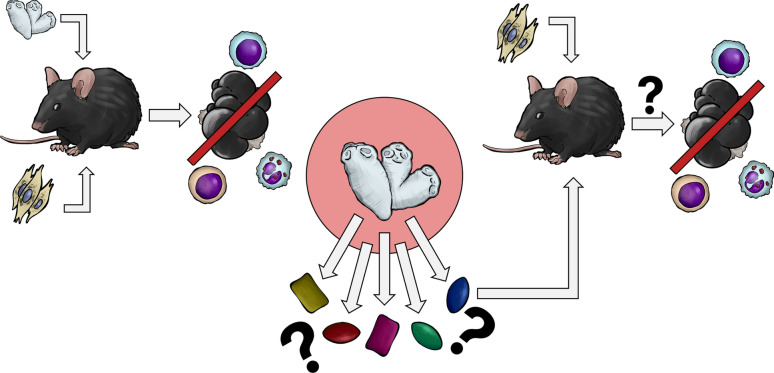

**Supplementary Information:**

The online version contains supplementary material available at 10.1186/s13071-026-07391-4.

## Background

Over the past two decades, observations have been made on the capability of certain parasites to ameliorate various human diseases via their immunomodulatory abilities [10, 63]. Specifically, tapeworms have been recently pointed to as agents that could help prevent or treat autoimmune diseases, such as type 1 diabetes [20], multiple sclerosis [56], or cancer [11]. The incidence of oncological diseases is continually on the rise [57], and with issues pertaining to their treatment being omnipresent [43], there is a high demand for new therapeutic approaches [67]. The natural cancer-suppressive effects of several tapeworm species have thus come under scrutiny [62], with the most studied species including *Echinococcus granulosus* [1], *Taenia crassiceps* [33], and more recently, *Mesocestoides corti* (Cestoda: Cyclophyllidea) [61].

*Mesocestoides corti* has a presumed three-host life cycle, where various carnivorous mammals serve as its definitive hosts. In a variety of secondary intermediate hosts (e.g., small rodents, birds, amphibians or reptiles), a metacestode, the tetrathyridium, develops in the liver and peritoneal cavity. This stage is unique in terms of its extensive asexual multiplication by longitudinal division [64]. These secondary larvae can be infinitely cultured/grown in experimental mice, which, along with their low propensity to infect humans, makes *M*. *corti* an ideal laboratory model for studies of tissue cestodiasis and interactions with host physiology and immunity [71, 73].

The primary immune response to an intraperitoneal infection with *M. corti* tetrathyridia is of the type 1 helper T cell type, which is essential for parasite destruction [27]. Importantly, the start of the infection is also characterized by the presence of CD8^+^ T cells and natural killer (NK) cells [36]. Later, a type 2 helper T cell response [72] is elicited by the parasite [5]. Specifically, alternatively activated macrophages play an important role in the suppression of a potentially pathogenic inflammatory immune response [41], which aids host survival [45].

In the case of *E. granulosus*, several anti-tumor mechanisms have been described, including the stimulation of immune cells by parasite antigens [44]. The direct effect of its excretory-secretory products (ESPs) was demonstrated through their ability to affect the proliferation of hamster fibrosarcoma cells [18]. Importantly, a specific molecule with cancer-suppressive capabilities was recently identified in this tapeworm. It is a Kunitz-type serine protease-specific inhibitor that suppresses the growth and migration of breast, cervical, and melanoma cancer cells in vitro via cell cycle disruption by inducing apoptosis. It was also able to significantly reduce the growth of breast cancer in mice, while not being cytotoxic to normal cells [53]. Moreover, it had the same in vivo effect on mouse melanoma [54].

Recently, *M. corti* infection also demonstrated a strong anti-cancer effect, as it mitigated melanoma growth and metastasis in mice [61]. While the infection caused a rise in potentially cancer-killing immune cells in the peritoneal site of infection. Here, the ESPs themselves showed no direct effect on cancer cells in vitro, hinting at an innate immunity-mediated suppression of this cancer cell line in mice, which warrants further investigation.

In order to discover possible effector molecules involved in the anti-cancer effect of *M*. *corti* ESPs, we analyzed the transcriptomes of tetrathyridia cultivated in vitro and obtained from inbred and outbred mouse strains. Proteins representing the theoretical secretome were identified by a bioinformatic search for signal sequences. Additionally, the total larval proteome and secretome of *M*. *corti* were analyzed by nano shotgun liquid chromatography with tandem mass spectrometry (MS–MS) (LC/MS–MS), together with label-free quantification. We compared our data to those of known tumor suppressors in related tapeworms, specifically *E. granulosus*, and attempted to identify candidates at both the bioinformatic and experimental levels in a new experimental model. An abundant Kunitz-type protease inhibitor [*M. corti* Kunitz-type chymotrypsin-specific inhibitor 1 (McKI-C1)], similar to the one with cancer-suppressive effect in *E. granulosus* [53], was produced in recombinant form. Its ability to affect cancer cells in vitro was evaluated using the B16F10 cell line, a highly aggressive, readily metastasizing variant of mouse melanoma [47], and ID8 ovarian carcinoma cell line (ID8), a relatively slower-growing cancer [23]. Both were chosen for their localization and use in our other in vivo experiments with tapeworm infections and ESPs applications. The in vivo effect of McKI-C1 on tumor growth and metastasis was assessed with B16F10 melanoma cells, since the *E. granulosus* Kunitz protease inhibitor has been successfully tested with a similar cell line.

## Methods

### Aims and design

 The aim of this study was to thoroughly investigate the transcriptome and proteome of the *M. corti* tapeworm, which possesses cancer-suppressive properties. The transcriptome was analyzed from *M. corti* larvae both directly after harvesting from either ICR or C57BL/6J (BL) mice and after in vitro culture, in order to assess any transcriptomic changes between the host environment, where cancer suppression occurs, and the cultivation process, which provides tapeworm homogenates and ESPs that could be potentially utilized in cancer suppression. 

The proteomic analysis was performed from both whole *M. corti* larval homogenate (McH) and concentrated ESPs of tapeworm larvae (*M. corti* larval excretory-secretory products; McES), in order to elucidate specific molecules, which are released into the host environment and have a potential effect on the murine immune system or could directly affect cancer cells. Upon the discovery of a highly expressed ortholog of an *E. granulosus* Kunitz-type protease inhibitor, i.e., McKI-C1, this protein was tested for its ability to abrogate cancer cell functions necessary for tumor growth and metastasis, both in vitro and in vivo. The former analysis examined the abilities of McKI-C1 to prevent cancer cell survival, growth, migration and extracellular matrix breakdown, while the latter looked at its ability to induce a reduction of the growth of B16F10 melanoma in mice, which infection with this tapeworm can achieve.

### Mice

The C57BL/6JOlaHsd inbred strain (female, aged 5 weeks) was supplied by Envigo and the Hsd:ICR (CD-1®) outbred strain (female, 11–15 g) was acquired from Charles River Laboratories. The mice were kept at the Centre for Experimental Biomodels (First Faculty of Medicine, Charles University). Housing was provided in conventional cages with a 12-h:12-h light:dark cycle at 23 °C with 55% humidity and ad libitum access to both water and feed. The total number of mice utilized in this experiment was 25. No animals were excluded during the experiment or during the data analysis. Mice were randomly allocated to treatment and control groups using computer-generated random numbers (in Excel). All authors handling the animals were aware of group allocation. Mice were sacrificed via anesthesia overdose (isoflurane inhalation).

### Parasite strain and infections of experimental animals

The isolate of *M*. *corti* was obtained from Dr. Ruth Fichter and Prof. Peter Deplazes (Institute of Parasitology, University of Zürich, Switzerland), where it has been maintained in mice for teaching purposes for many years. Tetrathyridia were passaged in female ICR mice (infection dose 600 larvae at age 6 weeks, harvested 3 months post-infection).

### Collection of parasite material for transcriptomic analyses

The tetrathyridia of *M. corti* were harvested from three female ICR mice 3 months post-infection with 600 larvae. The larvae were pooled and 600 were used to obtain each of three samples with the following methods: (1) in vitro cultivation for 14 days in high-glucose Dulbecco’s modified Eagle’s medium (DMEM) (Sigma-Aldrich), which was changed every other day, together with penicillin–streptomycin (100 U/ml), but no fetal bovine serum (FBS)] (IV samples); (2) intraperitoneal inoculation into ICR outbred mice (ICR samples); and (3) intraperitoneal inoculation into C57BL/6J inbred mice (BL samples). Each sample was prepared in four biological replicates. Following 2 weeks of incubation/parasite growth in mice, the tetrathyridia were collected and their total RNA was isolated with the use of TRIzol™ Reagent (Thermo Fisher Scientific) and treated with TURBO™DNase (Thermo Fisher Scientific).

### Library preparation and sequencing

In total, 12 libraries (3 × 4 replicates) were constructed by using NEBNext**®** Poly(A) mRNA Magnetic Isolation Module [51] and NEBNext**®** Ultra II Directional RNA Library Prep Kit for Illumina (NEB #E7760, #E7765) [52]. The libraries were pair-end sequenced on the NovaSeq platform (Illumina). Searchable sequence data for this study have been deposited in the European Nucleotide Archive (ENA) at the European Molecular Biology Laboratory-European Bioinformatics Institute (EBI) under accession number PRJEB97085 (https://www.ebi.ac.uk/ena/browser/view/PRJEB97085).

### Data preprocessing

Sequence reads were inspected for quality by FastQC version 0.11.5 [3]. Sequencing errors were corrected by Rcorrector version 1.0.4 [66] and low-quality reads were filtered by Trimmomatic version 0.38 [7], with the minimum length of reads being 25 base pairs.

### Annotation of reference transcriptome

The reference transcriptome of *M. corti* PRJEB510.WBPS15 [39] was functionally annotated using two complementary approaches. National Center for Biotechnology Information (NCBI)-based annotation was performed by sequence similarity searches against the NCBI non-redundant protein database (version March 2020 [59]) using Basic Local Alignment Search Tool + (BLAST+; version 2.10.1), with an *e*-value threshold of 1e−5 and a maximum of five target sequences per query. For each transcript, a single representative annotation was selected based primarily on functional relevance and secondarily on the highest bit score. In addition, Kyoto Encyclopedia of Genes and Genomes (KEGG) annotation was obtained using the GhostKoala automatic annotation server (version 2.2) with the KEGG database [28–30].

### Differential gene expression analysis

Cleaned and trimmed reads of IV, BL and ICR samples were mapped to the reference transcriptome to assess gene expression by RNA-Seq (RNA sequencing) by Expectation–Maximization (RSEM; version 1.3.3) [34], and transcripts with expected counts ≥10 in all replicates were considered as transcribed. Differentially expressed genes were identified by DESeq2 (version 1.30.1) [35] for each pair of samples (IV and BL, IV and ICR, ICR and BL). Genes with adjusted *p*-value < 0.05 were identified as differentially expressed and sorted to six groups according to the level of log2-fold change (log2FC), as follows: highly downregulated (log2FC < −2); downregulated (log2FC [−2, −1]); slightly downregulated (log2FC [−1, 0]); slightly upregulated (log2FC [0, 1]); upregulated (log2FC [1, 2]); and highly upregulated (log2FC > 2). Transcripts classified as slightly up- or downregulated (|log2FC|< 1) were included for descriptive purposes only and were not considered biologically relevant or interpreted further.

### Identification of *E. granulosus* Kunitz inhibitor 1 orthologs in the *M. corti* transcriptome

#### Homology search

Orthologs of the *E. granulosus* Kunitz-type protease inhibitor [*E. granulosus* Kunitz inhibitor 1 (EgKI-1); accession no. EUB56407.1] were identified in the *M. corti* reference genome (PRJEB510.WBPS15). The BLAST+ tool (version 2.12.0) [12] was employed for this purpose, using a stringent e-value threshold of 1 × 10^−5^. Expression levels of identified orthologs were derived from RSEM-based quantification as described above.

#### Transcriptome mapping and quantification

Gene expression levels were calculated from RSEM mappings and expressed as transcripts per million (TPM), providing a normalized metric suitable for comparative expression analysis across samples. Expression values for M. corti orthologs of EgKI-1 were calculated as the arithmetic mean of four biological replicates for each sample group (BL, ICR, and IV).

#### Multiple alignment of EgKI-1 and* M. corti* Kunitz protein orthologs

Identified *M. corti* orthologs were subjected to multiple sequence alignment using the Clustal Omega algorithm as part of the Job Dispatcher suite at the European Molecular Biology Laboratory-European Bioinformatics Institute [37]. The alignment was used to identify conserved cysteine residues and amino acids in the inhibitory P1 site, providing insights into functional and structural conservation.

### Verification of McKI-C1 sequence

Total RNA was isolated from tetrathyridia obtained from ICR mice by using TRIzol™ Reagent (Thermo Fisher Scientific) and reversely transcribed into complementary DNA by a QuantiTectReverse Transcription Kit (Qiagen). Complementary DNA served as a template for PCR production of the McKI-C1 coding gene (primers used—McKI_fw 5′-GGTGGTGGTGCTCGAGTCACGGTTAATCCTTGCACGC-3′, McKI_rev 5′-AAGGAGATATACATATCAGTGGTGATGGTGATGATGG-3′). Amplified products were cloned into pGEM®-T Vector System (Promega) and *E*. *coli* strain XL-1 blue cells. Clones were Sanger sequenced with M13 primers (DNA Sequencing Laboratory, BIOCEV, Vestec, Czech Republic). The verified sequence is available in GenBank under accession number PX254364. The signal sequence of the protein was determined with SignalP 6.0 (https://services.healthtech.dtu.dk/services/SignalP-6.0/). Potential N- and O-glycosylation sites were checked with NetNGlyc 1.0 (https://services.healthtech.dtu.dk/services/NetNGlyc-1.0/) and NetOGlyc 4.0 (https://services.healthtech.dtu.dk/services/NetOGlyc-4.0/), respectively. Prediction of generic phosphorylation sites was performed with NetPhos 3.1 (https://services.healthtech.dtu.dk/services/NetPhos-3.1/). Theoretical molecular weight and isoelectric point values were calculated with the Compute pI/Mw tool (Expasy; https://web.expasy.org/compute_pi/).

### Collection of parasite material for proteomic analyses

To obtain ESPs of tetrathyridia, the larvae were harvested from five intraperitoneally infected ICR mice 3 months post-infection, washed 5 times with sterile phosphate-buffered saline (PBS), placed in 75-cm^3^ culture flasks (Eppendorf® Cell Culture Flask T-75) in DMEM supplemented with 100 U/ml of penicillin–streptomycin and cultured at 37 °C, 5% CO_2_. The following day, the larvae were transferred into new flasks to remove the remaining adhered immune cells. The medium was collected and replaced every other day for 2 weeks, passed through a 0.22-μm sterile syringe-mounted filter (Sigma-Aldrich) and stored at −80 °C. The samples were concentrated with Amicon Ultra (molecular weight cut-off 10 kDa; Merck-Millipore) and the medium replaced with sterile PBS. Protease inhibitors were added (cOmplete ULTRA Tablets, Mini, EDTA-free, EASYpack Protease Inhibitor Cocktail; Sigma-Aldrich). Each of the four McES biological replicates was based on the secretions of approximately 900 tetrathyridia (1.5 ml).

For the preparation of *M*. *corti* homogenate, the aforementioned larvae were washed in sterile PBS, placed into a solution of protease inhibitors in sterile PBS and sonicated 3 times at 60 W for 30 s. After centrifugation at 16,000 *g* for 20 min, the supernatant was collected and sterile filtered. Two biological replicates (two different rounds of cultivation) of the soluble homogenate fraction (McH) were used for MS analysis.

### Nanoflow liquid chromatography–MS–MS of McH and McES

#### Protein digestion

The samples were lysed by boiling at 95 °C for 10 min in 100 mM triethylammonium bicarbonate (TEAB) containing 2% sodium deoxycholate, 40 mM chloroacetamide, and 10 mM Tris(2-carboxyethyl)phosphine and further sonicated (Bandelin Sonoplus Mini 20, MS 1.5). The protein concentration was determined by using a bicinchoninic acid (BCA) protein assay kit (Pierce BCA Protein Assay Kit; Thermo Fisher Scientific) and 10 µg of protein per sample was used for MS sample preparation. The sample volume was adjusted to 65 µl by adding 100 mM TEAB containing 2% sodium deoxycholate. Samples were further processed using SP3 beads on a KingFisher Flex automated Extraction & Purification System (Thermo) in a 96-well plate. Briefly, 65 µl of sample was added to 65 µl of 100% ethanol and mixed with the SP3 beads. After binding, the beads were washed three times with 80% ethanol. After washing, samples were digested in 50 mM TEAB at 40 °C with 1 µg of trypsin for 2 h, then another 1 µg of trypsin was added and the samples were left overnight. After digestion, the samples were acidified with trifluoroacetic acid to 1% final concentration and peptides were desalted using in-house-made stage tips packed with C18 disks (Empore) [55].

#### Nano LC/MS–MS

Nano reverse phase columns (Aurora Ultimate TS 25 × 75 C18 ultra-high-performance liquid chromatography column; Ion Opticks) were used. Mobile phase buffer A was composed of water and 0.1% formic acid. Mobile phase B was composed of acetonitrile and 0.1% formic acid. Samples were loaded onto the trap column (5-μm particle size, 300-μm × 5-mm C18 PepMap100; Thermo Scientific) for 4 min at 18 μl/min. The loading buffer was composed of water, 2% acetonitrile and 0.1% trifluoroacetic acid. Peptides were eluted with mobile phase B gradient from 4 to 35% B in 60 min. Eluting peptide cations were converted to gas-phase ions by electrospray ionization and analyzed on a Thermo Orbitrap Ascend (Thermo Scientific) by a data-independent approach. Survey scans of peptide precursors from 350 to 1400 m/z were performed in Orbitrap at 60-K resolution (at 200 m/z) with a 4 × 10^5^ ion count target. DIA scans were performed in Orbitrap at 30-K resolution. The AGC target was set to 1000% and the maximum injection time mode to auto. Precursor mass range 400–1000 m/z was covered by thirty 20-Da-wide windows. Activation type was set to HCD with 28% collision energy.

### Proteomic data analysis

The MS proteomics data have been deposited in the ProteomeXchange Consortium via the PRIDE [49] partner repository, with the dataset identifier PXD068152. All of the data were analyzed and quantified with the Spectronaut 19 software [9], using directDIA analysis. The data were searched against the *M*. *corti* database (PRJEB510, WBPS15, downloaded from WormBase in March 2025, containing 22,215 entries). C-terminal enzyme specificity was set to Arg and Lys, which also allowed for cleavage at proline bonds and a maximum of two missed cleavages. Carbamidomethylation of cysteine was set as a fixed modification and N-terminal protein acetylation and methionine oxidation as variable modifications. The false discovery rate was set to 1% for peptide-spectrum match, peptide and protein. Quantification was performed at the MS2 level. Precursor posterior error probability cutoff and precursor and protein cutoff were set to 0.01. Protein posterior error probability was set to 0.05. Data were exported and data analysis was performed using Perseus 1.6.15.0 [70].

### Protein annotation

The identified proteins were annotated using the non-redundant NCBI database (BLASTp; ; *E*-value cut-off 10^−5^) [12] and KEGG [28], using the GhostKoala tool (version 2.2) [30] (BLASTp; *E*-value cut-off 10^−5^). Additional functional annotation was performed with InterPro [6], using InterProScan version 5.74–105.0. Reliably identified proteins in McES were further functionally annotated according to Gene Ontology (GO) terms [4, 68] based on InterPro domain identifiers, using the InterProGO mapping file downloaded from the Gene Ontology Consortium website (https://current.geneontology.org/ontology/external2go/interpro2go) in June 2025. GO terms were categorized into the three main ontologies—biological process, molecular function and cellular component-according to the structure defined in the go-basic.obo ontology file and parsed using the GOATOOLS Python package. For each GO term, the name, GO category, and associated transcript identifiers were retrieved and summarized for downstream analysis and visualization.

### In silico identification of *M*. *corti* secretome

To identify proteins secreted via the classical pathway, amino acid sequences derived from the reference transcriptome of *M. corti* (PRJEB510, WBPS15) were analyzed using SignalP version 6.0 with parameters optimized for eukaryotic organisms. Proteins predicted to contain an N-terminal signal peptide were retained for further filtering. To exclude membrane-associated proteins, we analyzed these SignalP-positive sequences using TMHMM version 2.0, which predicts transmembrane helices and topologies. Only proteins lacking predicted transmembrane domains (i.e., with zero predicted transmembrane helices) were retained as high-confidence secretome candidates.

### Gene enrichment analysis

Differentially expressed transcripts were mapped to KEGG pathways using KEGG Mapper (version 5.0). Overrepresentation of KEGG pathways among up- and down-regulated transcripts was assessed using Fisher’s exact test. Pathways with *p*-values ≤ 0.05 were considered significantly enriched. All data processing and enrichment analyses were performed using custom Python scripts.

### Preparation and purification of recombinant McKI-C1

*Mesocestoides corti* Kunitz protein with the highest level of gene transcription (MCU_012597-RA, termed “McKI-C1”) was chosen for recombinant expression and functional studies. The sequence of the McKI-C1 gene without the part coding for a signal sequence was codon-optimized in silico for expression in *E*. *coli*; the gene was synthesized and cloned into the pET-22b(+) vector in an open reading frame with a pelB leader sequence and with 6× His-Tag at the C-terminus (GenScript, Netherlands). The Rosetta-gami B(DE3)-competent *E*. *coli* strain (Novagen) was transformed with the construct by heat shock in accordance with the supplier’s instruction manual. The selection of positive clones was performed on Luria-Bertani (LB) agar plates with 0.3 mM ampicillin (Amp). For protein production, the cells were first grown in 10 ml liquid LB medium plus 0.1 mM Amp at 37 °C, 230 r.p.m. for 6 h. The bacterial suspension was transferred into 1 l LB medium plus 0.3 mM Amp with 20 mM glucose and incubated in baffled flasks at 37 °C, 230 r.p.m., overnight (final optical density ≈ 2). The cells were centrifuged and transferred into 1 l fresh LB medium plus 0.05 mM Amp and induced with 1 mM IPTG for 20 h at 16 °C, 230 r.p.m.. Cells frozen in lysis buffer (20 mM Tris–HCl, 0.3 M NaCl, 40 mM imidazole, 1% lauroylsarcosine, pH 8.0) were thawed, sonicated for 10 min at 6-s cycles and 20 W (Vibra Cell™ 72405; Bioblock Scientific). 

Two-step purification of McKI-C1 was performed on Äkta pure 25 L1 (Cytiva) apparatus using (1) a HisTrap FF column (GE Healthcare) for immobilized metal affinity chromatography, and (2) a MonoS column (GE Healthcare) for cation-exchange chromatography at pH 6. Purified protein was concentrated on Amicon Ultra-15 filters (molecular weight cut-off, 3000; Merck), transferred into PBS, pH 7.2 (20 mM phosphate buffer, 150 mM NaCl), and potential contamination by bacterial endotoxin was prevented by using a Pierce High-Capacity Endotoxin Removal Spin Column (1.0 ml; Thermo Fisher Scientific). The final protein solution was sterile filtered, aliquoted and stored at −20 °C. The protein concentration was determined with the BCA-1 assay kit (Merck). 

Purification steps were monitored by sodium dodecyl sulfate-polyacrylamide gel electrophoresis (SDS-PAGE) on 8–16% TGX gradient gels (Bio-Rad). The theoretical molecular weight and isoelectric point of recombinant McKI-C1 were calculated using the Expasy Compute pI/Mw tool (https://web.expasy.org/compute_pi/). The identity of McKI-C1 after SDS-PAGE was confirmed by MS of in-gel digested tryptic fragments [26]. Conserved amino acid residues of the putative serine protease binding site on conserved Kunitz-type domain were identified by NCBI Conserved Domain Search [42, 76].

### Inhibition tests with recombinant McKI-C1

The functionality of purified recombinant McKI-C1 was tested with four proteases and corresponding aminomethylcoumarin-coupled synthetic fluorogenic peptide substrates Suc-Ala-Ala-Pro-Phe-7-amino-4-methylcoumarin (AMC) (Suc-AAPF-AMC) (Bachem) or Z-Gly-Gly-Arg-AMC (Z-GGR-AMC) (Merck) [25]. Bovine α-chymotrypsin (10 nM final concentration; catalogue no. C6423; Merck), cathepsin G from human leukocytes (15 µM; 219373; Merck), elastase from porcine pancreas (4 µM; E1250; Merck), and urokinase from human urine (100 nM; 672112; Merck) were mixed with various concentrations of McKI-C1 in reaction buffer (50 mM HEPES, 150 mM NaCl, 5 mM CaCl_2_, 0.05% Tween-20; the buffer for urokinase was free of calcium) in a total volume 50 µl per well of a black flat-bottom microtitration plate (Nunc), and left to stand for 10 min at room temperature. Then, 50 µl of the respective substrate solution in the reaction buffer was added (final concentration 50 µM) and the relative fluorescence units of released AMC were measured in an Infinite M200 fluorometer (Tecan, Austria) at 355/460 excitation/emission wavelengths, at 25 °C for at least 30 min in 1- or 2-min kinetic cycles. The half-maximal inhibitory concentration (IC_50_) of McKI-C1 (final concentrations 1000, 500, 100, 50, 10, 5, 1, and 0.1 nM) with chymotrypsin was calculated from three measurements and plotted in GraphPad Prism version 10.2.0. The results were expressed as relative peptidolytic activity in the well, with McKI-C1 related to the activity of the enzyme alone taken as 100% (Δ relative fluorescence units/min, linear part of the curve). For cathepsin G, urokinase and elastase inhibition, the concentrations of McKI-C1 were 4 and 0.4 µM, and the results are shown as rough output graphs from single measurements.

### Cell culture

Mouse melanoma B16F10 cells obtained from ATCC (CRL-6475) and mouse ovarian carcinoma ID8 cells obtained from Sigma-Aldrich (SCC145) were cultured in standard conditions (37 °C, 5% CO 2) in complete Dulbecco’s modified Eagle medium (DMEM) (Life Technologies) with 4.5 g/L L-glucose, L-glutamine, and pyruvate, supplemented with 10% FBS (Merck) and 0.1% ciprofloxacin (Sigma-Aldrich).

### Toxicity

An Alamar Blue assay (Invitrogen) was performed to assess the cytotoxicity of McKI-C1. Cells were seeded in 96-well plates (SPL Life Scientific) at 1 × 10^4^ (B16F10) and 2 × 10^3^ (ID8) cells in either complete medium (DMEM plus 10% FBS and ciprofloxacin), or complete medium with McKI-C1, at a concentration of 46 µg/ml. After 72 h, the medium was aspirated and 50 µl of Alamar Blue solution (10% Alamar Blue reagent in complete medium) was added for 4 h. Afterwards, fluorescence was measured using an Infinite M200 PRO fluorescent plate reader (Tecan) at 560/590 nm. Three independent experiments were performed with 10 wells for each cell line, either treated or control.

### Wound healing

Cells [5 × 10^4^ (B16F10) or 3 × 10^4^ (ID8)] were seeded in 96-well plates (Sartorius). The following day, a scratch was made across the well with a Incucyte 96-well Woundmaker Tool (Sartorius). The cells were washed twice and either complete medium (DMEM plus 10% FBS and ciprofloxacin), complete medium with McKI-C1 at 4.6 or 46 µg/ml, or complete medium with latrunculin A (Merck) was added, the latter serving as a negative control. The plates were transferred into an Incucyte S3 microscope and imaged every 2 h for a total of 24 h. The healed area was calculated using Incucyte Scratch Wound Analysis Software (Sartorius) as relative wound density, which is shown in the graph as a mean of 10 wells for each condition and cell line.

### Proliferation

For the kinetic measurement of growth, cells were seeded in 96-well plates (SPL LifeScientific) at 1 × 10^4^ (B16F10) or 2 × 10^3^ (ID8) cells, in either complete medium (DMEM plus 10% FBS and ciprofloxacin), or complete medium with McKI-C1 at a concentration of 46 µg/ml. The plates were then transferred into an Incucyte S3 microscope and imaged every 4 h for a total of 72 h. Cell proliferation was calculated from the images as an increase in cell area using Incucyte Basic Analysis software, which is shown in the graphs as a mean of 20 wells over two independent experiments for each cell line, with each well imaged in four areas.

### Gelatin degradation assay

A gelatin degradation assay was performed as per the manufacturer’s instructions (QCM TM Gelatin Invadopodia Assay, Merck Millipore). Cells were resuspended in either complete medium (DMEM plus 10% FBS and ciprofloxacin) or complete medium with McKI-C1 at 46 µg/ml. Subsequently they were seeded on gelatin for 16 h, fixed and then stained with Alexa Fluor 405 Phalloidine (ThermoFisher Scientific) to visualize actin and 4′,6-diamidino-2-phenylindole for nuclei. Fluorescent images were acquired with a Nikon T-E H-TIRF microscope equipped with a Nikon 60×/1.49 NA oil objective. The gelatin degradation was quantified by scoring the area of degradation as a percentage of total cell area, with 40–50 cells scored for both treatment and control conditions by using ImageJ software.

### In vivo experimental design

Two groups of seven BL female mice were intraperitoneally inoculated with 3 × 10^5^ B16F10 cells in sterile PBS at the age of 8 weeks. One week later, an inoculation regimen was started, whereby one group was inoculated with 80 µg of McKI-C1 in sterile lipopolysaccharide-free PBS and the other with lipopolysaccharide-free PBS only as a control. The mice received inoculations every other day for a total of seven doses each over a period of 2 weeks. Subsequently, the mice were sacrificed, blood for serum was collected, peritoneal lavage was performed, and the melanoma tumors were excised and weighed. The lungs, liver and spleen were also retrieved.

### Positive control sera for enzyme-linked immunosorbent assay

A group of five 6-week-old female BL mice were individually intraperitoneally injected with 600 *M. corti* tetrathyridia. The mice were sacrificed 35 days later and their blood collected and serum separated from it.

### Enzyme-linked immunosorbent assay

The determination of McKI-C1-specific immunoglobulin M (IgM) and immunoglobulin G (IgG) antibodies in mouse sera was performed in 96-well plates (Nunc MaxiSorp, Thermo Fisher Scientific), which were coated with either McKI-C1 or McH at 2.5 ng/µl, diluted in a carbonate-bicarbonate buffer at pH 9.6 and incubated overnight at 4 °C. Following a washing step, the plates were blocked with 5% non-fat dried milk in PBS for 1 h at 37 °C, then washed again and incubated for 2 h at room temperature with sera diluted in the blocking solution at 1:500 for IgM and 1:100 for IgG. After another wash, goat anti-mouse secondary antibodies with conjugated peroxidase [anti-mouse IgM antibody (Abcam ab97230), anti-mouse IgG fragment crystallizable-specific antibody (Sigma-Aldrich A2554)] were added, diluted to 1:10,000 for anti-IgM or 1:8000 for anti-IgG in 0.5% bovine serum albumin in PBS and incubated at room temperature for 2 h. Following the final wash, peroxidase substrate (Tetramethylbenzidine—liquid substrate system for enzyme-linked immunosorbent assay; Sigma-Aldrich) was added. The reaction was then stopped with 2 M H_2_SO_4_. Absorbance values were read at 450 nm. Cut-off values were determined in accordance with Frey et al. [21], using the measurements from uninfected mice.

### Isolation and immunophenotyping of peritoneal cavity cells

The isolation of peritoneal leukocytes was performed by washing the peritoneal cavity with 10 ml of ice-cold sterile PBS. The cells were centrifuged for 10 min at 350 *g* at 4 °C, and erythrocytes lysed with ammonium-chloride-potassium buffer. The remaining cells were then washed in PBS, and cell counts were determined using a Countess® II Automated Cell Counter (Thermo Fisher Scientific). Afterwards, the cells were incubated with an anti-CD16/CD32 antibody for 20 min at room temperature and stained with Zombie Aqua™ Fixable Viability Kit at 1:600 dilution. Following a wash with 3% FBS in PBS, the cells were incubated with a mixture of surface marker antibodies, as specified in Fig. S1, for 30 min at 4 °C. Measurements were performed on a CytoFLEX flow cytometer (Beckman Coulter) and analyzed in FlowJo (version 10.8.1). Fluorescence minus one control use is specified in Fig. S1 and the representative gating strategy in Fig. S2.

### Statistical analysis

The statistical analysis of transcriptomic and proteomic data is described in detail in the respective sections above. Quantitative data were visualized and analyzed in GraphPad Prism (version 10.5). The correlation analysis of average log2 abundance and expected transcript counts was also performed in GraphPad Prism. Values obtained from individual mice are shown along with the group average and SD. The specific tests that were applied are indicated in the figure legends. *p*-values < 0.05 were considered to indicate statistical significance; The exact *p*-value is shown if it is the range from 0.05 to 0.10.

Relative inhibition of chymotrypsin by McKI-C1, along with IC_50_, was analyzed using non-linear regression. Inhibition of cathepsin G, and elastase, by McKI-C1 was analyzed by repeated-measures two-way ANOVA. Cytotoxicity and gelatin degradation by cancer cells under the effect of McKI-C1 were analyzed with an unpaired *t*-test. Proliferation and wound healing were analyzed with a mixed-effects two-way ANOVA. Peritoneal melanoma tumor weight, mouse body weight and spleen-to-body weight ratio, as well as cell counts in the peritoneal immune cell milieu, were analyzed by an unpaired *t*-test.

## Results

Due to *M. corti*’s cancer-suppressive capabilities, which we have previously described, we decided to perform a transcriptomic and proteomic analysis of its products in order to explore its potentially immunomodulatory molecules. Having discovered an ortholog of EgKI-1, which we have termed “McKI-C1,” and that might have the ability to prevent the growth and metastasis of cancer due to its similarity to EgKI-1, we described and recombinantly produced the former in order to test its function as a protease inhibitor, as well as its in vitro and in vivo abilities to suppress cancer.

### Transcriptomic analysis of *M. corti* larvae from mice and in vitro cultivation

**Fig. 1:**
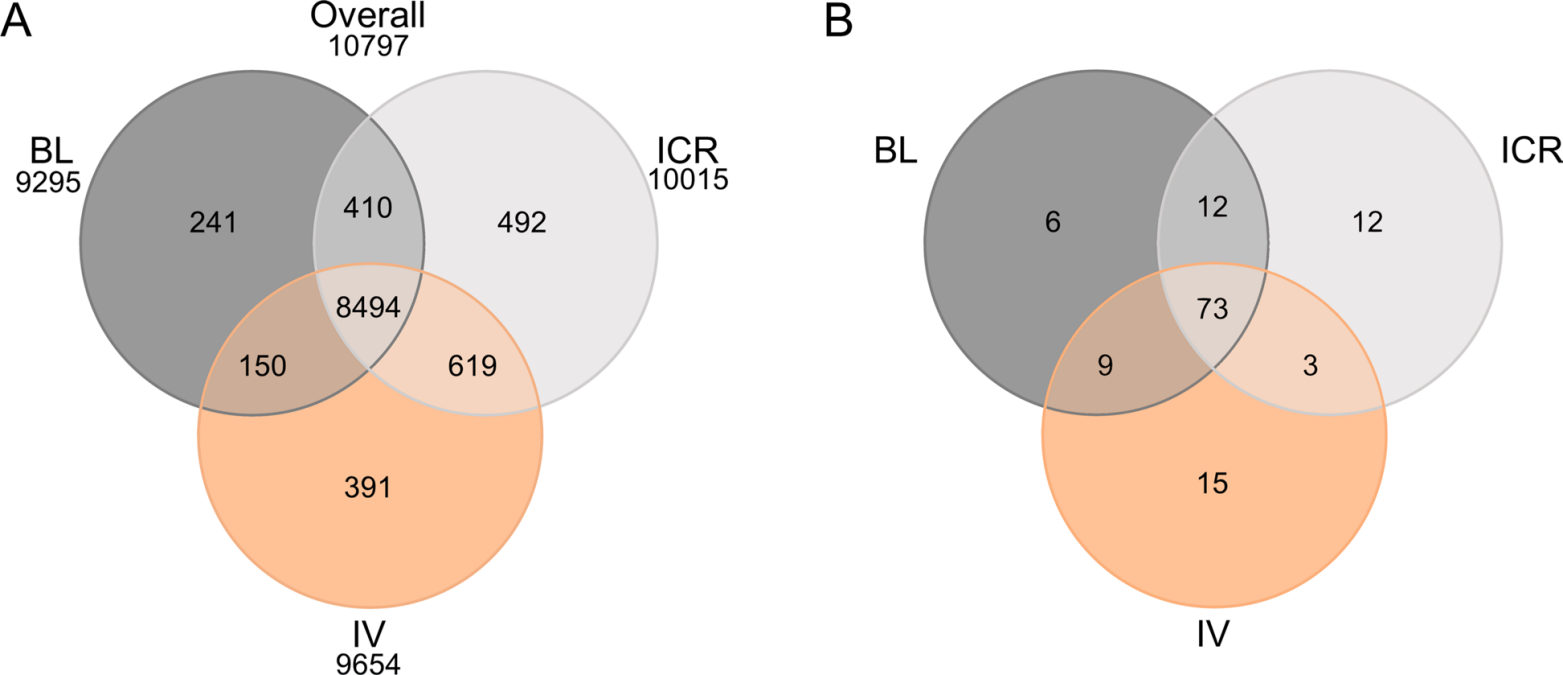
Venn diagrams of transcribed protein-coding genes and the 100 most transcribed protein-coding messenger RNAs (mRNAs) per group. **A** Number of transcripts unique to, or common to, specific groups. The vast majority of transcripts are shared. **B** Number of the 100 most transcribed proteins that are unique to, or common to, specific groups

The transcriptomic profiles of *M. corti* larvae either harvested from BL or ICR mice or larvae collected after a 14-day cultivation period were compared (Fig. 1A). In total, 10,797 transcripts were identified, of which 9295 (86%) were found in BL (from BL mice), 10,015 (93%) in ICR (from ICR mice) and 9654 (89%) in IV samples. By comparing the samples, we found that there were 241 (2.23%) unique transcripts in the BL group, and 492 (4.56%) in the ICR group. In the IV group, 391 (3.62%) unique transcripts were found. Transcripts found only in BL and ICR totaled 410 (3.8%), while those found only in the ICR and IV groups totaled 619 (5.73%). There were 150 transcripts (1.39%) that were common only to the BL and IV samples. In total, 8494 transcripts were common to all three groups, and constituted 78.67% of all of the transcripts.

### The most highly expressed protein-coding transcripts in *M. corti*

**Fig. 2:**
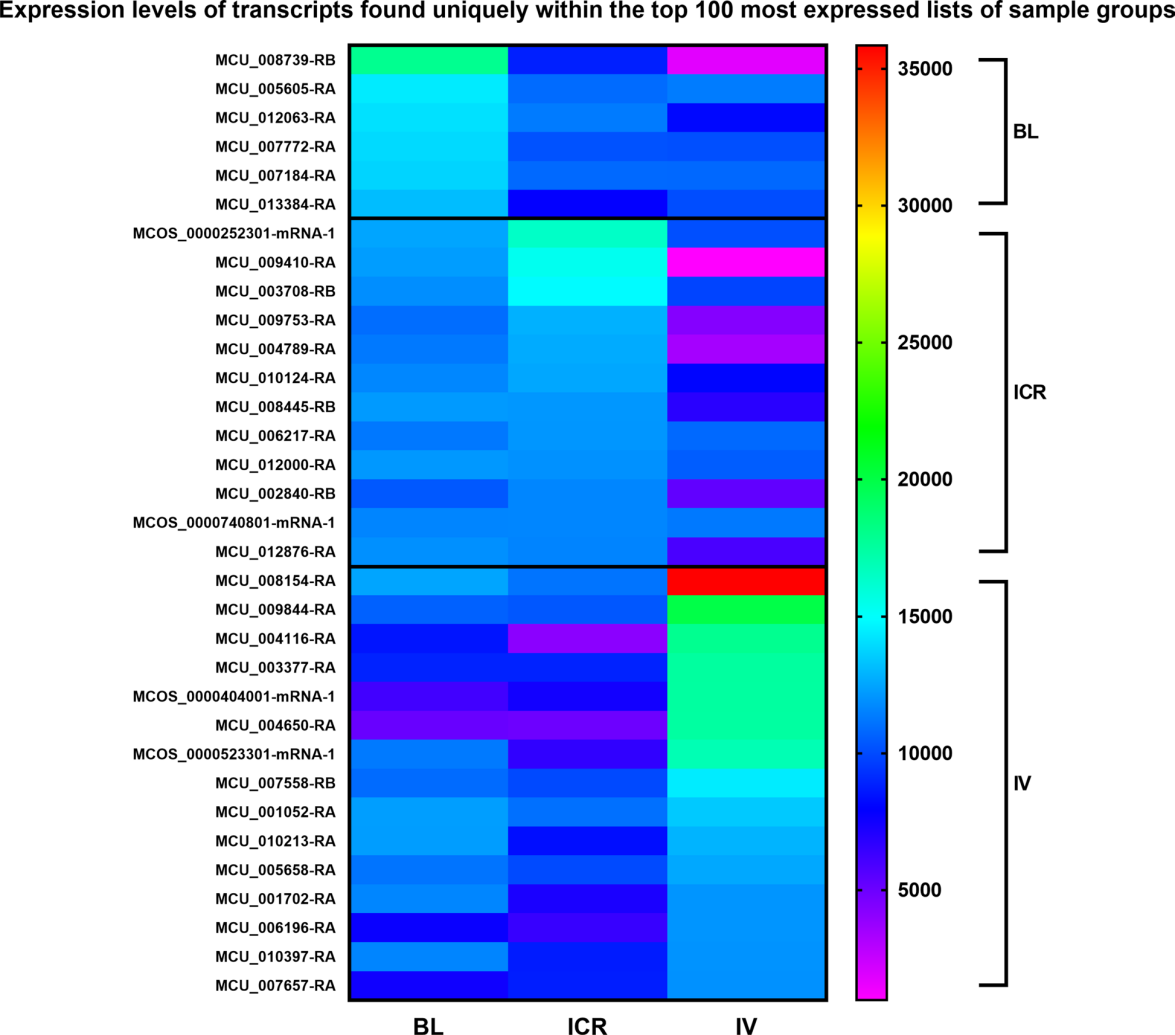
Heat map showing the expression levels of transcripts found uniquely within the top 100 most transcribed protein-coding mRNAs per group. The graph shows the expression levels of transcripts that were found uniquely within the top 100 most transcribed protein-coding mRNAs, which are listed for each group [C57BL/6J mouse tetrathyridia group (BL), ICR, in vitro cultivated tetrathyridia group (IV)], while also showing their expression levels in the other samples

In order to ascertain the overlap of the most transcribed protein-coding genes, the expected transcript counts were averaged across all samples for each group. A list of 100 transcripts with the most expected counts was made (Supplementary Table 1). The lists of these transcripts were compared to see how much they differ across groups (Figs. 1B, 2). While 33 of these 100 transcripts were unique [six to BL, 12 to ICR and 15 to IV (in vitro samples)], they were often (19 of them) within the top 200 transcripts of the other groups, meaning that they were also highly transcribed, but only barely made the list, e.g., the transcript MCU_005605-RA ranks amongst the top 100 in only the BL group, and ranks 112th and 102nd in the lists for ICR and IV, respectively. The positions of these transcripts dropped when comparing mouse groups with IV, with the greatest difference being that of the presence of a unique top-100 ICR transcript at position 1549 in IV. Respective listings of the transcripts in the other groups are also shown in Supplementary Table 1. These lists were searched for an important group of protein products, the superfamily of cysteine-rich secretory proteins [CAP; also annotated as glioma pathogenesis-related (GLIPR1-like), venom allergen-like (VAL), cysteine-rich secretory proteins, etc.], which is explored further in the proteomics section below. There were nine members of this superfamily in the BL list, 10 in the ICR list, and four in the IV list.

### Transcriptional differences between in vivo- and in vitro-derived *M. corti*

KEGG pathway-based gene enrichment analysis revealed clear functional differences between the in vivo- and in vitro-derived tapeworms. Parasites isolated from both inbred (BL) and outbred (ICR) mouse hosts predominantly showed enrichment of metabolic and energy-related pathways, including general metabolism, biosynthesis of secondary metabolites, and oxidative phosphorylation, indicating elevated metabolic activity under in vivo conditions. In contrast, in vitro-cultivated parasites exhibited enrichment of pathways associated with environmental information processing and cellular stress responses, including mitogen-activated protein kinase, Ras, and Notch signaling, as well as pathways related to translation, lysosomal function, autophagy, and apoptosis. KEGG categories annotated as human disease–related pathways were not further interpreted, as they likely reflect broadly conserved cellular processes rather than biologically relevant disease mechanisms in this context. The complete results of the KEGG enrichment analysis are provided in Supplementary Tables 2 and 3. In addition, complete results of the differential expression analyses, including NCBI- and KEGG-based functional annotations, are provided in Supplementary Tables 4, 5 and 6.

### Host strain-dependent differential gene expression in *M. corti*

Beyond the pronounced transcriptional differences between in vivo and in vitro conditions, additional variation in gene expression was observed among parasites isolated from different mouse host strains. Transcriptomic comparison of *M. corti* recovered from ICR and BL mice revealed extensive host strain–dependent differences across a broad spectrum of functional categories. Parasites isolated from BL mice exhibited increased expression of genes associated with transcriptional regulation, intracellular signal transduction, ubiquitin-mediated protein turnover, and vesicular trafficking. These included transcription factor 7-like 2, a key effector of the Wnt/β-catenin pathway (TCF7L2; MCU_008376-RA), and the regulatory subunit of phosphoinositide-3-kinase (PIK3R1; MCU_011791-RA). Additional upregulated genes encoded DDB1- and CUL4-associated factor 12, involved in ubiquitin-mediated protein regulation (DCAF12; MCU_000686-RB), and a Rab9 effector protein with kelch motifs implicated in intracellular vesicular transport (RABEPK; MCU_000473-RC).

In contrast, parasites obtained from ICR mice showed higher expression of genes associated with membrane-associated signaling, vesicular sorting, and lipid and glycerophospholipid metabolism. These included P2X purinoceptor 4, a ligand-gated ion channel involved in purinergic signaling (*P2RX4*; MCU_003006-RA), vacuolar protein sorting–associated protein 13A, a component of vesicular trafficking machinery (*VPS13A*; MCU_005708-RC), sterol O-acyltransferase, involved in lipid metabolism (*SOAT*; MCU_005290-RD), and glycerophosphodiester phosphodiesterase 1, which participates in glycerophospholipid metabolism (*GPCPD1*; MCU_001792-RA). In addition, several transcripts exhibited very high fold changes despite relatively low absolute expression levels, including genes associated with mitogen-activated protein kinase-related signaling, ubiquitin-mediated protein turnover, and membrane-associated functions. Together, these results indicate that host genetic background influences not only genes involved in fundamental cellular processes in *M. corti*, but also the deployment of distinct parasite strategies in response to host-specific environments. A complete list of significantly differentially expressed transcripts identified by DESeq2, together with their functional annotations, is provided in Supplementary Table 4. Notably, a substantial proportion of the genes upregulated in parasites isolated from ICR mice encoded secreted or surface-associated proteins, prompting a more detailed analysis of immunomodulatory gene families, which is discussed below.

### Differential expression of immunomodulatory gene families in parasites isolated from ICR and BL mice

Among the transcripts upregulated in parasites recovered from the BL mice, genes encoding members of immunomodulatory protein families were particularly prominent. These included multiple representatives of the CAP (cysteine-rich secretory proteins, antigen 5, and pathogenesis-related 1) superfamily, such as peptidase inhibitor 16 (*PI16*; MCU_001595-RA and MCU_011681-RA), cysteine-rich secreted protein 2 (MCU_004110-RA and MCU_006136-RA), cysteine-rich secreted protein 3 (MCU_004157-RB), and a CRISPLD-domain–containing protein (MCU_011041-RA). Elevated expression was also observed for a CAP superfamily protein, VAL2 (MCU_012951-RA), further highlighting the enrichment of secreted and surface-associated molecules in this host background.

Collectively, these findings demonstrate that host strain–specific conditions not only fine-tune global transcriptional programs in *M. corti*, but also strongly influence the expression of parasite gene families that are directly involved in the modulation of host immune responses.

### Proteomic analysis of *M. corti* larval secretome and homogenates

**Fig. 3:**
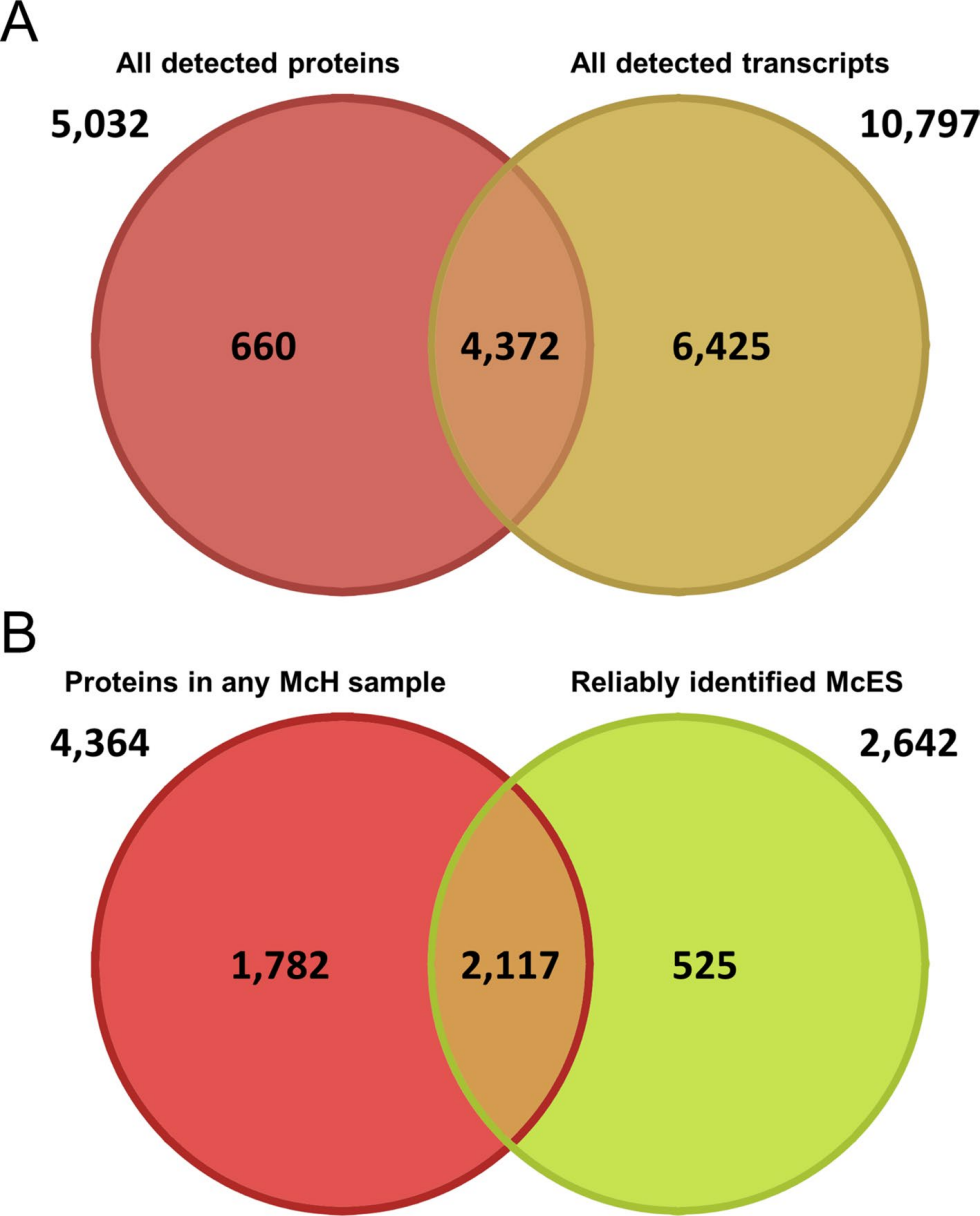
Venn diagrams of unique and common proteins within samples of *Mesocestoides corti*. The diagrams show the proteins that were also detected in the transcriptome (**A**) and also compare the proteins that are common to both samples, and those that are unique to each sample (**B**)

**Fig. 4:**
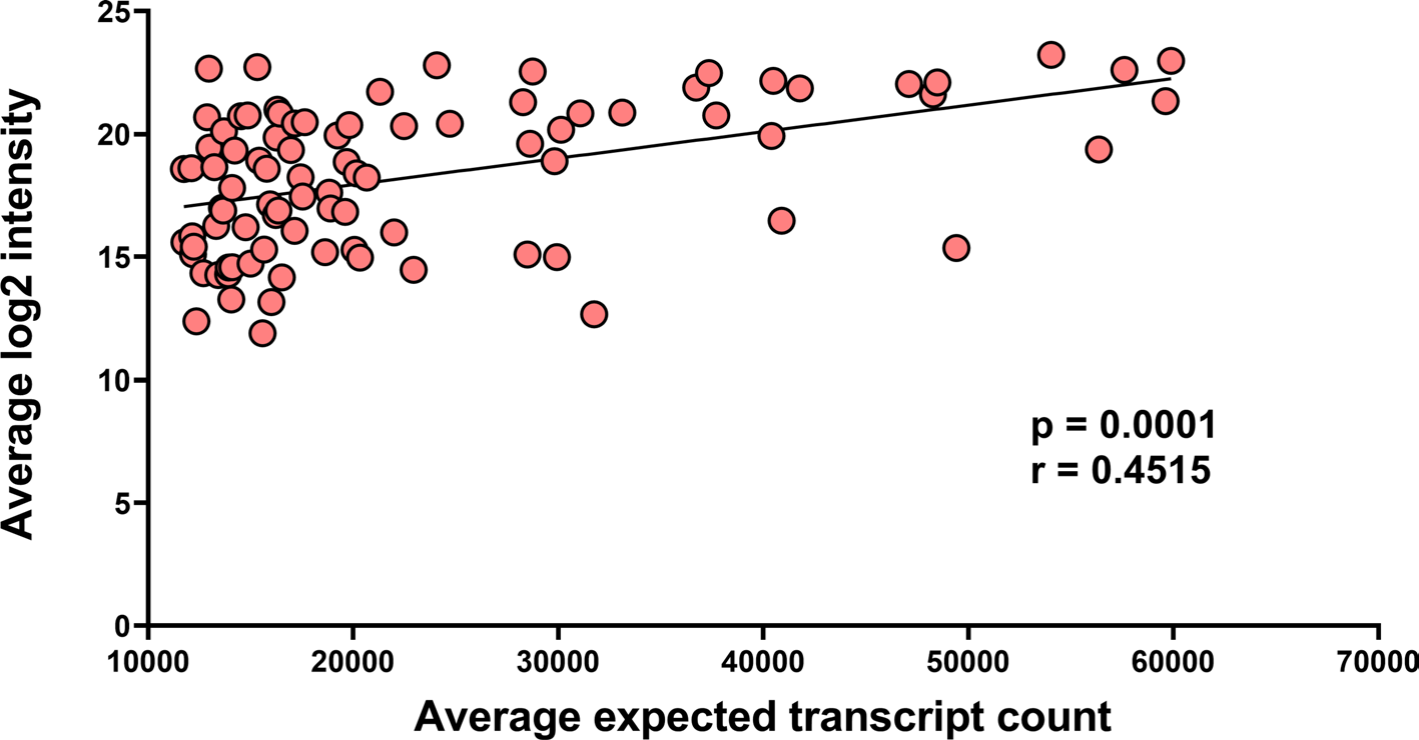
Correlation of protein abundance and expression levels of the top 100 most abundant protein**s.** The graph shows that the proteins that show higher abundance in the proteome also show higher transcription levels in the transcriptome

The protein contents of *M. corti* ESPs collected during 14-day ex vivo cultivation and larval homogenates prepared after 14 days of cultivation were analyzed. By means of MS–MS, we identified 55,809 unique peptides (i.e., stripped sequences) in total. Among these, 32,681 and 46,121 were detected in at least one sample of McES and McH, respectively. The list of identified peptides, their modifications and accompanying data are presented in Supplementary Table 7. When mapping the peptides to the predicted proteins of *M*. *corti* (22,215), they were assigned to 5032 proteins in total (Fig. 3), of which 4372 (86.88%) were also detected as transcripts in tetrathyridia cultivated either in vitro (IV) or harvested from BL or ICR mice (Supplementary Table 1). The total number of proteins found in any McES sample was 3250, and 4364 proteins were found in any McH sample (1782 were McH unique). For the overall results of the proteomic analysis and protein annotations, see Supplementary Table 8. Among all the McES proteins, we considered 2642 to have be detected in at least three out of four reliably identified McES (riMcES) samples. Of these, 525 were uniquely detected in McES and 2117 were shared with McH. Only 329 of all of the identified proteins (6.54%) occurred in the “hypothetical” in silico secretome inferred from the tapeworm’s total genome-derived in silico transcriptome (based on the predictions of signal peptides in SignalP and transmembrane domains in TMHMM). Detailed statistics, annotations and results of the GO analysis of riMcES can be found in the Supplementary Table 8 (N.B. use the filters as needed for each column). Overall, when correlating the average protein abundance and the average expression levels of their transcripts, proteins with higher abundance in the proteome also showed higher expression levels in the transcriptome (Fig. 4).

After Interpro functional annotation of riMcES, GO analysis was performed, and terms were assigned to particular proteins, where possible (Supplementary Table 8). Only 1304 (49.4%) of these were assigned ≥ 1 GO terms—696 hits were retrieved from the superior category biological process, 368 from cellular component and 1397 from molecular function. The frequencies of occurrence of particular riMcES protein-associated GO terms within the principal categories are summarized in Supplementary Table 9.

**Fig. 5:**
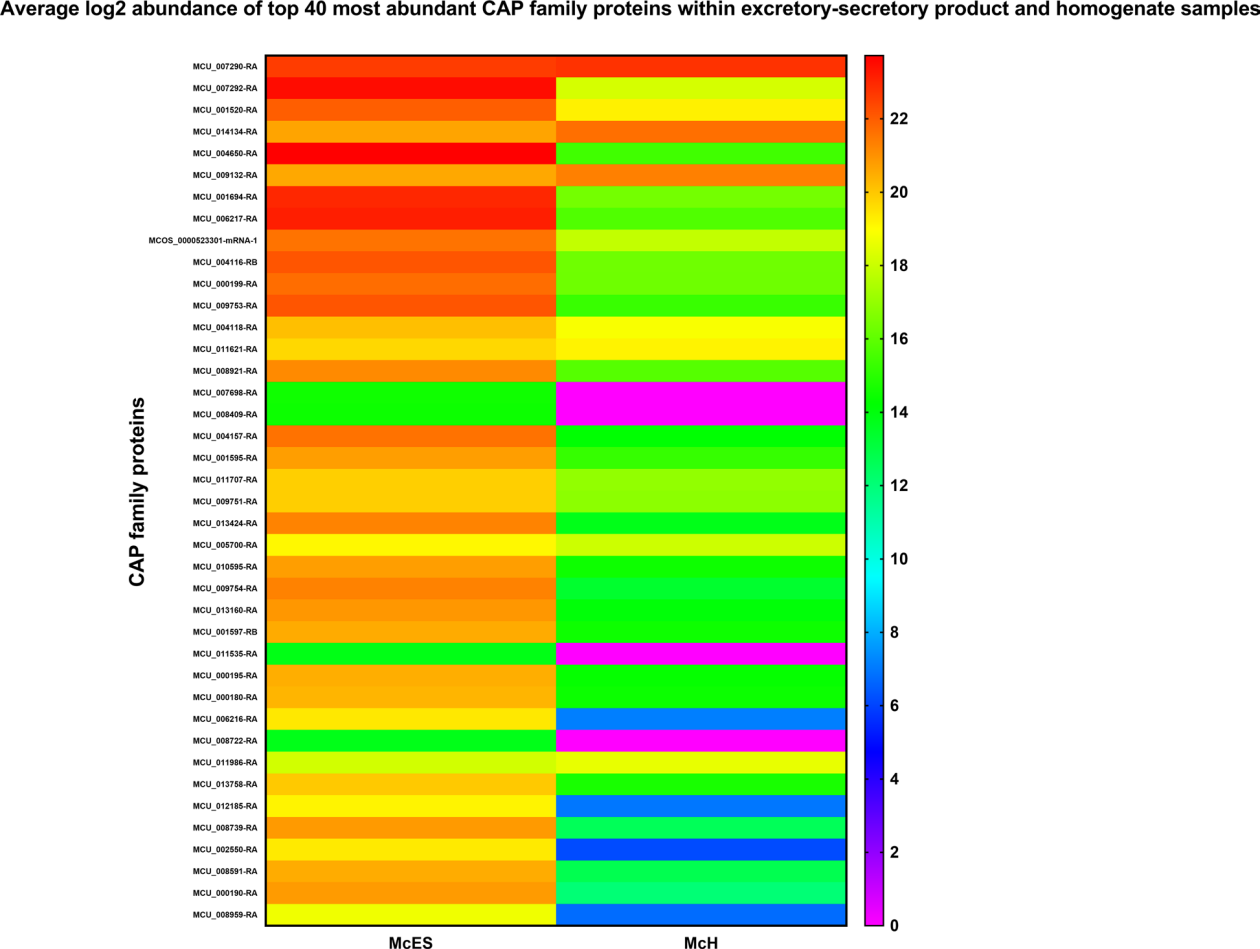
Heat map showing the abundance of the 40 most abundant CAP family proteins found within the proteome of *Mesocestoides corti* excretory-secretory products (ESPs). The graph shows the average log2 abundance of the 40 most abundant CAP family proteins averaged across the *M. corti* larval excretory-secretory products (McES) as well as their average abundance in the *M. corti* whole larval homogenate (McH) samples

**Fig. 6:**
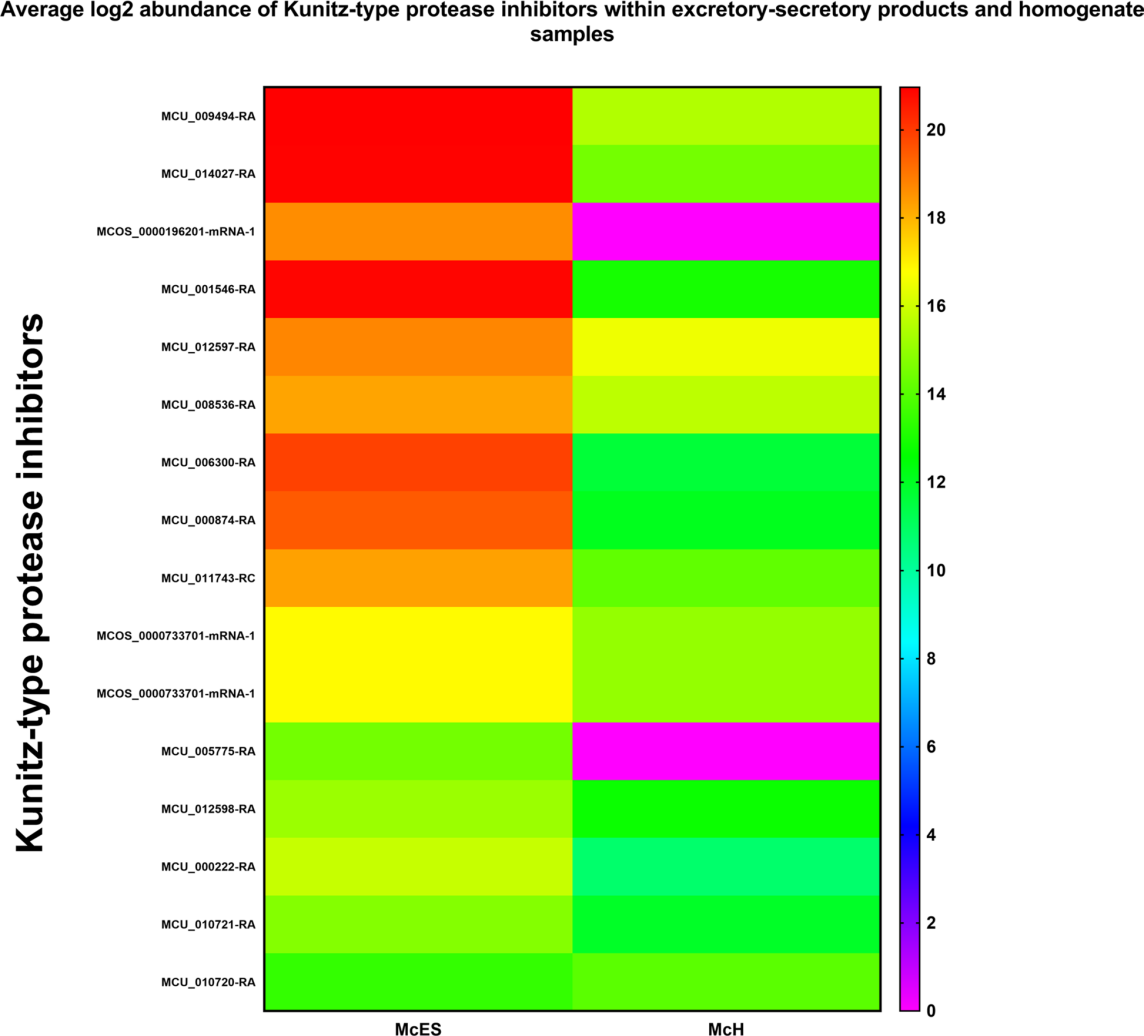
Heat map showing the abundance of Kunitz-type protease inhibitors found within the proteome of *Mesocestoides corti*. The graph shows the average log2 abundance of Kunitz-type protease inhibitors averaged across both McES and McH samples. For abbreviations, see Fig. 5

There were 23 unknown proteins among the riMcES, with no NCBI or Interpro hits, and another 129 proteins annotated as an “unnamed protein product” of *M*. *corti*. Some of these occurred even among the most abundant proteins in riMcES. According to the annotations, the samples also contained cytoplasmic and membrane proteins, likely contaminants from the tetrathyridia. The normalized quantification showed average label-free quantification intensities among riMcES, ranging from ca. 2 × 10^7^ to 7 × 10^2^ (N.B. use the filter to sort by McES average intensity value in Supplementary Table 7). The most conspicuous group of proteins in terms of abundance—either the number of representatives (coded by unique transcripts) or quantitative proportion among riMcES—was the CAP superfamily, 168 of which were found (Fig. 5), largely without GO terms and description (with very few exceptions). Nine of these occurred among the first 50 most abundant proteins, and five of them among the first 20. In most of them, a secretion-targeting signal sequence was predicted in silico. Filtering out possible non-secretory protein contaminations (i.e., considering only riMcES proteins with a predicted signal sequence and no predicted transmembrane domain), 29 CAP superfamily proteins occurred in the first quartile of the most abundant proteins (making up ≈39% of the protein species). Another functionally important group of proteins, richly represented in riMcES, included peptidase inhibitors (some of which were among the first 50 most abundant proteins). We identified 16 Kunitz-domain inhibitors (Fig. 6), 11 annexin family proteins, six cystatins, and five serpins in total. A number of proteolytic enzymes included mainly nine cysteine endopeptidases (cathepsins L and B, calpains), four serine family S1 trypsin-like endopeptidases and two aspartic family A1 endopeptidases. Metallopeptidases were represented mainly by several leucyl aminopeptidases of the M17 family.

### Orthologs of EgKI-1 were found in *M. corti*

**Fig. 7:**
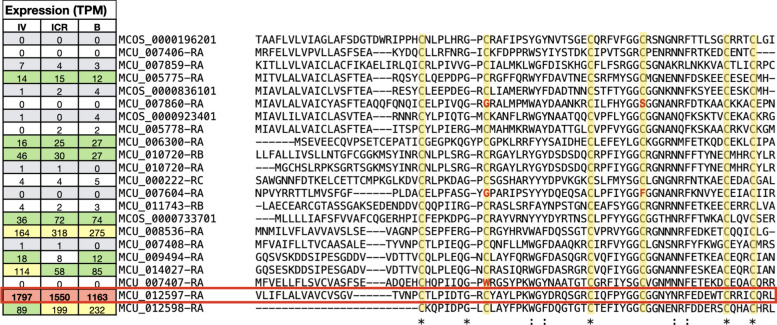
Multiple alignment of partial amino acid sequences of *Mesocestoides corti* orthologs with the Kunitz-type protease inhibitor from *Echinococcus granulosus* (EgKI-1; accession no. EUB56407.1)]. Conserved cysteine residues are highlighted in yellow, with substitutions marked in red. Amino acids in the P1 position, which determine protease inhibition specificity, are enclosed by a green box. The level of gene transcription for each ortholog is given as transcripts per million (TPM) in a table for each sample type: in vitro cultivated, ICR, and BL mice. Transcription values represent the arithmetic mean of four biological replicates, with conditional formatting applied to indicate transcription levels. The ortholog with the highest gene transcription is enclosed by a red box

A total of 22 different orthologs of EgKI-1 were identified within the reference transcriptome of *M. corti*. Among these, 17 sequences were found in the transcriptomic data from the IV, BL, and ICR sample groups. Multiple sequence alignment (Fig. 7) revealed conserved cysteine residues characteristic of Kunitz-like protease inhibitors. Specifically, 19 orthologs exhibited the typical six conserved cysteine residues, two displayed four, and one had five of them. Expression levels were calculated for all orthologs and ranged from 1 to 1797 TPM. The most expressed ortholog, *MCU_012597-RA*, demonstrated TPM values of 1797, 1550, and 1163 in the IV, ICR, and BL samples, respectively. Furthermore, a tyrosine residue was identified in the P1 site of *MCU_012597-RA*, indicative of chymotrypsin inhibitory activity (Fig. S3). The total length of the preprotein is 91 amino acids, including a signal sequence of 19 amino acids. The theoretical molecular weight of the pre-protein/mature protein is 10,378/8386 Da, and its pI 7.51/6.31. Neither N- nor O-glycosylation sites were predicted in silico. Potential predicted phosphorylation sites occur at Thr12, Ser28, Thr70 and Ser65 (mature protein numbering). We termed the protein McKI-C1 (Fig. S3) in this study “*Mesocestoides corti* Kunitz inhibitor, chymotrypsin-specific, no. 1.”

### Recombinant McKI-C1 inhibits chymotrypsin-like proteases

**Fig. 8:**
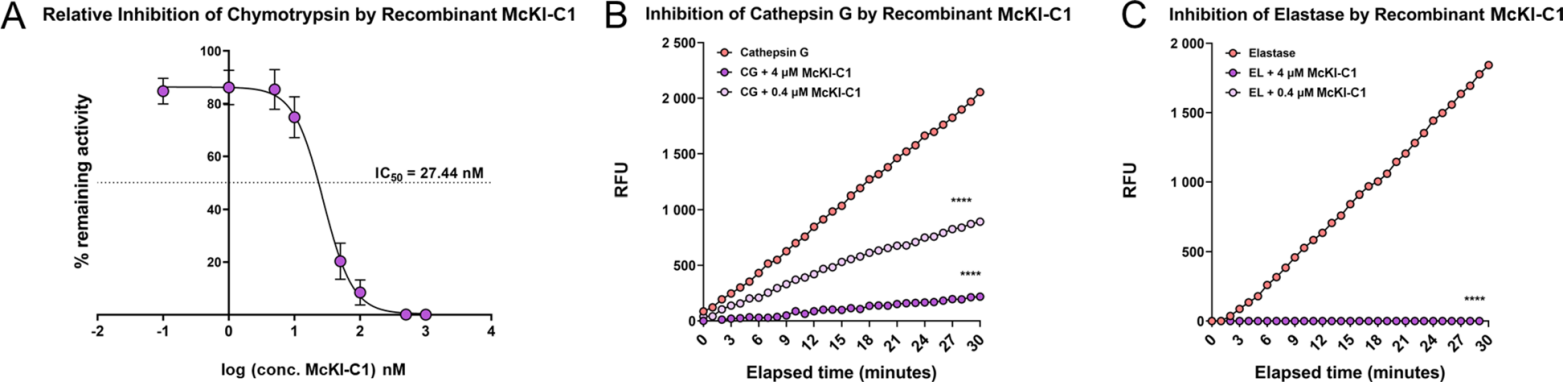
Relative inhibition of chymotrypsin, inhibition of cathepsin G and inhibition of pancreatic elastase by the recombinant Kunitz-type chymotrypsin-specific inhibitor 1 from *Mesocestoides corti* (McKI-C1). **A** The graph shows the relative inhibition of chymotrypsin. The dashed line indicates the half-maximal inhibitory concentration (IC_50_) of chymotrypsin activity. Relative inhibition values are related to the activity of chymotrypsin, which was considered to be 100%. Suc-Ala-Ala-Pro-Phe-7-amino-4-methylcoumarin (AMC; 50 µM) fluorogenic substrate was used. The IC_50_ values were extrapolated from three measurements by non-linear regression. **B** The graph shows the inhibition of cathepsin G (CG), with kinetic data from a measurement using 15 µM cathepsin G and 50 µM Suc-Ala-Ala-Pro-Phe-AMC fluorogenic substrate. *RFU* Relative fluorescence units. The statistical analysis was undertaken with repeated-measures two-way ANOVA (****p < 0.0001). **C** The graph shows the inhibition of pancreatic elastase (EL), with kinetic data from a measurement using 4 µM elastase and 50 µM Suc-Ala-Ala-Pro-Phe-AMC fluorogenic substrate. Statistical analysis by repeated-measures two-way ANOVA (****p < 0.0001)

To test the specificity of McKI-C1 towards serine peptidases and its possible tumor-suppressive activity, the protein was produced in *E*. *coli* and purified to homogeneity, as assessed by SDS-PAGE (Fig. S4). MS analysis confirmed its identity and purity (Fig. S3). Its calculated molecular weight is 9,340.45 Da, including the 6× His tag and N-terminal methionine, and the theoretical pI is ≈ 7. All tested serine peptidase family S1 enzymes (chymotrypsin-like) were inhibited by McKI-C1 (Fig. 8A–C), while urokinase activity was not inhibited (data not shown). Since the peptidolytic activities of commercially supplied porcine elastase and cathepsin G towards the substrate were much lower (approximately 2 and 3 orders of magnitude, respectively, related to the molar concentrations of the enzymes) than that of chymotrypsin, it was not possible to calculate and reliably compare the IC_50_ of McKI-C1 for the enzymes, except for chymotrypsin (IC_50_ = 27.44 nM) (Fig. 8A).

### In vitro effect of McKI-C1 on B16F10 melanoma and ID8 ovarian carcinoma cells

In order to ascertain the capabilities of McKI-C1 to directly affect cancer cells, its effect on B16F10 melanoma and ID8 ovarian carcinoma was analyzed. Using an endpoint assay (Alamar Blue) along a kinetic holographic microscopy-based assay, the cytotoxicity of McKI-C1 to these cell lines was assessed. It showed no effect on cell survival for either cell line (Fig. S5A), while it showed some suppressive effect only on B16F10 proliferation, but none on ID8 (Fig. S5B). Using a scratch-wound assay, the effect of McKI-C1 on cell migration was analyzed; no difference was shown in the cells’ ability to close the wound (Fig. S5C, D). Lastly, the ability of these cell lines to degrade gelatin when treated with McKI-C1 was tested; however, no significant change compared to the control was found (Fig. S5E, F).

### McKI-C1 inoculation does not induce the suppression of B16F10 melanoma tumor growth

**Fig. 9:**
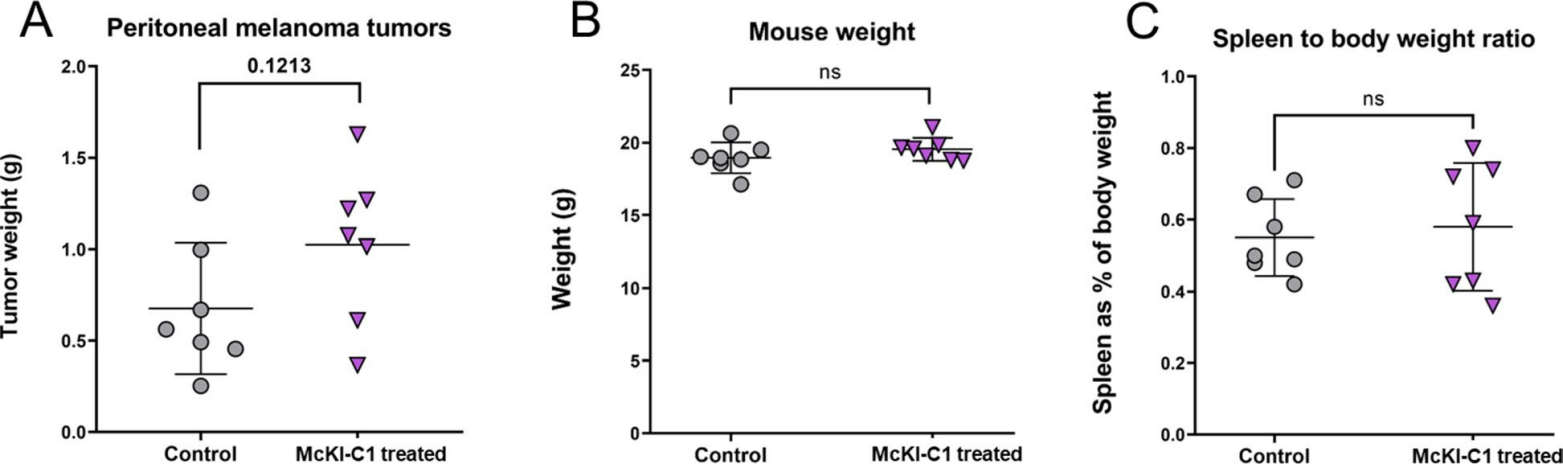
In vivo effect of McKI-C1 treatment on the growth of peritoneal melanoma cell line B16F10 (B16F10). The weight of excised melanoma tumors from the peritoneal cavities of mice (*n* = 7) showed no significant suppressive effect of McKI-C1 treatment (**A**). There was also no difference in mouse body weight when comparing the two groups (**B**). Neither was there a difference in spleen-to-body weight ratio (**C**). All statistical analyses were made by using an unpaired *t*-test. *Ns* No significant difference

In order to assess the ability of treatment with McKI-C1 to affect cancer growth, all of the melanoma tumors from the peritoneal cavity were excised and weighed 3 weeks post-inoculation of B16F10 cancer cells, with treatment being applied for the latter 2 weeks. No significant difference in overall weight, when compared with McKI-C1-treated and control mice, was found (Fig. 9A). Mouse body weight, as another measure of tumor growth severity, also showed no difference between the groups (Fig. 9B). Spleen-to-body weight ratio was also assessed as a measure of systemic adaptive immune response; however, no differences were found (Fig. 9C). To check for an antigen-specific immune response, McKI-C1-specific serum antibodies (IgM and IgG) were measured, but none were detected in McKI-C1-treated mice or in *M. corti*-infected mice (Fig. S6A, B). In order to explore the peritoneal immune milieu, the overall numbers of peritoneal leukocytes were counted, along with analysis of activation markers; however, there was no significant difference found in any of the cell counts measured (Fig. S7).

## Discussion

In our previous study [61], *M. corti* peritoneal infection successfully suppressed the growth and metastasis of B16F10 melanoma in mice, suggesting a potentially immune-mediated mechanism. In order to evaluate possible parasite candidate molecules involved in anti-cancer mechanisms, it was necessary to obtain an overview of proteins that are actually expressed by the tetrathyridia. These in vivo experiments were performed with BL mice, but the tapeworm larvae were kept in a laboratory cycle in ICR mice due to their larger size and lower cost. Furthermore, ESPs and tapeworms for homogenate production were obtained from in vitro cultivation. Thus, we decided to compare the transcription profiles of the larval tapeworms from inbred (BL) and outbred (ICR) mice, as well as from in vitro cultivation. We identified 10,797 transcripts in total, which represent ≈48.5% of the 22,265 messenger RNA transcripts present in the WBPS15 *M. corti* reference transcript set [24]. This proportion reflects the subset of annotated transcripts that were actively expressed under the experimental conditions examined. Our analysis was based on mapping reads to the reference transcriptome followed by conservative count-based filtering, an approach commonly used to reduce technical background noise and to provide a robust basis for transcript quantification and subsequent differential expression analysis [15]. Of the detected transcripts, 78.7% were common to all three sample groups, and 82.5% were common to both mouse strains. Therefore, we assume that utilization of the outbred ICR mice to maintain the parasite cycle, and the larvae produced in this way for subsequent production of ESPs and worm homogenates in vitro, are suitable and reproducible procedures for obtaining material for the exploration of the anti-cancer effect discovered primarily in BL mice.

It is quite common for the abundance of proteins (translation level) in a sample to not follow the level of transcription, i.e., actual RNA abundance [74]. Therefore, we employed proteomic approaches to thoroughly characterize the *M*. *corti* larval secretome (McES) and proteome of soluble fractions from larval homogenates (McH). To achieve this, we took advantage of the shotgun analysis of the complex samples by LC/MS–MS. The total number of protein species found in soluble McH was 4364, which highly exceeds the number of proteins identified in previous studies: 66 by Laschuk et al. [32], 115 by Vendelová et al. [71, 73], and 238 by de Lima et al. [19]. Such a vast difference in the number of identified proteins was most likely due to the rapidly increasing sensitivity of MS methods. Thus, we can assume that the present study provides, to the best of our knowledge, the most comprehensive information on the proteins of *M*. *corti* tetrathyridia, including quantitative data, to date.

Since the McES of tetrathyridia and not the larval homogenates have been found to be often responsible for known interactions with the host, including, for example, the suppression of dendritic cell functions [71, 73], we analyzed their protein composition by nano LC/MS–MS, too. The number of riMcES (total 2642) highly exceeds the number of proteins identified in larval McES to date [i.e., 55 proteins reported by Vendelová et al. [71, 73]) which, we believe, is clearly due to the more precise method employed in our study (shotgun proteomics vs. the excision of bands from the gel after one-dimensional electrophoresis) and the use of McES that had been collected over a relatively long period of time. The riMcES proteins were thoroughly annotated by employing NCBI, InterPro, KEGG and GO, since the returned annotations/terms did not always overlap and, in some cases, no hits at all were returned from a particular database. After summarizing the annotations, we decided not to use GO enrichment analysis, since fewer than half of the proteins had been assigned a GO term. This was due to the presence of proteins with unknown or uncertain functions in the proteome, which had no association with a GO term.

Comparing the “real” secretome (riMcES) with the in silico (SignalP and TMHMM) predicted total *M. corti* secretome inferred from translated coding genomic sequences, we found that 294 (ca. 11.6%) of the riMcES proteins have no transmembrane domain and possess a signal peptide targeting them to the conventional secretory pathway. Although this seems to be a low proportion of the proteins detected in the riMcES, there is a high probability that many of the proteins with no predicted signal peptide can be secreted via unconventional mechanisms, including by exosomes. However, on examination of the annotations, we cannot exclude the presence of some cytoplasmic or membrane proteins in the McES samples that may have originated from sporadically occurring dead or damaged tetrathyridia. On the other hand, those 294 riMcES proteins with predicted signal peptides and no transmembrane domain do constitute 61.2% of the total 482 *M. corti *in silico-predicted secretory proteins (genome sequence derived).

Almost 20% of the riMcES proteins were unique to these samples and did not occur in the homogenates. This seems counter-intuitive, since the secreted proteins must be synthesized within the bodies of the larvae and, therefore, should be present in them. However, the actual content of a particular secretory protein in the body may not have been sufficiently high for its detection, even by the sensitive method used here. On the other hand, secretions of the larvae were collected continuously for 14 days, pooled and filter-concentrated, which may have resulted in an increase in the amounts and proportions of secretory proteins in these McES samples, and thus increased their detectability. However, McES were collected throughout the entire cultivation period, but McH only on day 14, and the production of certain McES proteins may have already stopped at later points during cultivation due to the tapeworm lacking unspecified stimulating host factors. Therefore, as we lack snapshot data of the McH before day 14 of cultivation, we were unable to detect these proteins in our McH samples. Such a shift in the composition of *M. corti* ES products through a prolonged period of cultivation has been reported [71, 73].

Various types of protease inhibitors were found among the most abundant proteins in the McES. Since proteases are very important in orchestrating the immunity of vertebrates, and play a crucial role in the regulation of tumor-immune cell symbiosis [48], their inhibition can modulate these interactions. Several exogenous protease inhibitors have indeed been confirmed to be potent tumor suppressors, including some of parasite origin (e.g., [13, 38, 54]). In McES, these proteases belong to the most abundant proteins, and, in the present study, we identified an even broader spectrum than previous studies [71, 73]. Having discovered a highly expressed Kunitz-type protease inhibitor, McKI-C1, in the *M. corti* transcriptome and secretome, we aimed to examine whether it possesses this type of suppressive potential.

A recombinant form of McKI-C1 was therefore produced in *Escherichia coli*, and its ability to inhibit selected proteases was tested in order to confirm its activity (or correct folding) and specificity. First, chymotrypsin, used as a representative of the S1 family of serine proteases, was efficiently inhibited by McKI-C1, with an IC_50_ of 27.44 nM at 10 nM enzyme concentration. Cathepsin G and elastase, which have been shown to play important roles in immune reactions involving granulocytes (eosinophils, neutrophils) or in tumor initiation, growth, and metastasis [14], were also inhibited by McKI-C1. On the other hand, the activity of urokinase (a plasminogen activator), which is overexpressed in many invasive types of cancer cells [2, 40], was not affected.

In order to assess the ability of McKI-C1 to affect cancer cells in vitro, several hallmark parameters of cancer growth were chosen. As EgKI-1 caused apoptosis in certain cancer cell lines while also inhibiting their migration, the experiments were designed to analyze its cytotoxic effects and capability to abrogate the proliferation of cancer cells, which are both crucial in managing cancer as a disease [16]. At the same time, disrupting the ability of cancer cells to metastasize [65] is equally important, because the spread of uncontrollably proliferating cells into other tissues is one of the main reasons for cancer lethality [8, 50]. The motility of cancer cells may be affected by Kunitz-type protease inhibitors [60]. This was assessed through the changes in their ability to close a scratch-made wound within a culture, and their propensity to break down the surrounding extracellular matrix when treated with McKI-C1. The latter was examined by assessing the extent of degraded collagen around solitary cells. The chosen protein concentration of McKI-C1 paraleled that used in EgKI-1 [53], being increased tenfold in the case of wound-healing following the initial failure of McKI-C1 to abrogate migration. McKI-C1, however, did not have an effect on any of these parameters, be it the survival of cells, their proliferation, migration, or ability to degrade collagen, save for the proliferation of the B16F10 line. Similar results were reported with whole *M. corti* ESPs [61, 62], which shows that the products themselves likely do not directly affect the cancer cells.

However, the suppressive effect of McKI-C1 in *M. corti* infections is demonstrable. Therefore, an immune-mediated mechanism was not ruled out, also because treating mice with certain *E. granulosus* peptides increased the cytotoxicity of their splenocytes, causing a rise in activated NK cells [44]. A McKI-C1-treatment regimen was performed that used the same conditions as in our previous studies [61, 62], where mice were infected with *M. corti* 2 weeks prior to intraperitoneal B16F10 introduction and sacrificed after another 3 weeks. In the present study, the mice received McKI-C1 injections similarly to the procedure reported in Ranasinghe et al. [54]. In the present study, B16F10 tumors were allowed to establish for 1 week, after which 2 weeks of treatment was undertaken. Continuous treatment was chosen due to the infeasibility of direct intra-tumor injections because of the disseminated form of peritoneal B16F10 growth. The McKI-C1 treatment did not show any effect on tumor growth (despite slightly suppressing B16F10 proliferation in vitro), mouse weight or spleen size. Flow cytometry also revealed that there were no changes in the numbers of leukocytes examined. The treated mice did not produce any McKI-C1-specific antibodies either. On the other hand, the *M. corti* infection did cause a reduction in melanoma growth, while also elevating the numbers of peritoneal NK cells, which are important effectors in cancer immunotherapy [58]. Therefore, it seems that, although promising when compared to the *E. granulosus* Kunitz-type protease inhibitor (that possesses common inhibitory effects towards certain proteases), McKI-C1 is likely not an effector molecule in infection-mediated cancer suppression. However, it is not yet clear, even in the case of EgKI-1, which part of the protein is responsible for the anti-cancer effects, i.e., whether a canonical inhibition of certain protease(s) highly expressed by tumor cells explains these, or if another part of the molecule (outside the Kunitz active site) is involved. Therefore, without undertaking further experiments it is hard to assess whether other *M. corti* Kunitz-like proteins from the set of 22 found in the transcriptome, which are also significantly less expressed than McKI-C1, both in vivo and in vitro, are better candidates for this type of study.

The most conspicuous highly represented and abundant protein species among riMcES were of the CAP superfamily, previously named SCP/TAPS (sperm-coating proteins—Tpx-1, Ag5, PR-1, and Sc7 family) (Pfam accession no. PF00188). This is a diverse group of cysteine-rich proteins with a conserved domain that evolved miscellaneous, yet mostly unknown or not fully understood, functions. They are involves in various biological processes in essentially all organisms. They are generally secreted proteins that are coded by genes that expanded in the genomes of many organisms, including important species of parasitic flatworms like schistosomes [31, 75] and cestodes, e.g., *M*. *corti*. Members of the CAP superfamily are widely recognized as key effectors at the host–parasite interface and have been implicated in immune modulation, tissue interaction, and parasite survival across diverse helminth species [22]. The consistent upregulation of these genes in parasites isolated from BL mice, together with their generally higher absolute expression levels compared to many regulatory transcripts, suggests enhanced immunomodulatory effects in this host strain. In *M. corti*, a strikingly large expansion of the CAP superfamily happened, which is represented by 271 members with complete domains [17]. We found 168 of them (≈62%) among the secreted proteins, and many were highly abundant in the samples. Surprisingly, Vendelová et al. [71, 73] identified only two CAP superfamily proteins in McES in their limited proteomic study. It is pointless to speculate about their possible functions without experimental data, since their functions greatly vary. They have been shown to be involved in, for example, developmental regulation, reproduction, tumor suppression and immune regulation in animals, including parasites [46]. Functional analysis of mouse and human GLIPR1, which is a member of this superfamily, showed growth suppression and proapoptotic activities in multiple cancer cell lines. GLIPR1 also stimulated an immune response that resulted in specific anti-tumor cytotoxic T-lymphocyte activities in a prostate cancer model [69]. Considering that numerous CAP superfamily proteins in the secretions of *M. corti* share homology with GLIPR1, these would be good candidates for further experimental testing of the anti-tumor activities demonstrated in McES in our recent, thus far unpublished, experiments.

The fact that whole *M. corti* ESPs appear to successfully abrogate cancer growth and metastasis in vivo but not in vitro indicates that, among the plethora of proteins produced by the larvae of this tapeworm, both during infection and in vitro cultivation, there are potential cancer-suppressive agents. The investigation of their cellular immunity-stimulating properties, which might be of potential use in the future, warrants further research. Overall, we have thoroughly examined the transcriptome and proteome, and established a comprehensive molecular resource, while the apparently promising protein McKI-C1 has been ruled out as an effector molecule in the tapeworm’s anti-tumor capabilities. Most importantly, a repertoire of *M. corti* products has been unveiled, among which there may be molecules of potential medical interest.

## Conclusions

The transcriptomic analysis of *M. corti* larvae from both in vivo and in vitro environments allowed us to ascertain the viability of using cultured products of McH and McES in further experiments, and showed little difference in the transcriptomic profiles of larvae directly acquired from mice and those from in vitro cultivation. Furthermore, we identified a theoretical in silico secretome and pinpointed transcripts with secretory sequences. The proteomic analysis—to the best of our knowledge, the most extensive one performed for this organism thus far—allowed us great insight into the various protein products of this tapeworm. This mainly enabled us to analyze the actual extensive secretome from collected ESPs, so that we can explore the broad list of potentially immunomodulatory molecules from the repertoire of this cancer-suppressive tapeworm. We identified a high number of proteins from disparate protein families, which prompts further research. The in vivo and in vitro tests of a Kunitz protein candidate, McKI-C1, showed that it has no effect on the murine immune system or on the cancer cells directly, although an orthologous molecule found in *E. granulosus* does. Therefore, McKI-C1 does not seem to be an effector among *M. corti*’s cancer-suppressive capabilities, with its likely immune-mediated effect remaining, thus far, molecularly unresolved. However, the transcriptomic and proteomic analyses unveiled a wide spectrum of potential immunomodulatory proteins and, since infection with *M. corti* significantly reduced B16F10 growth, further study into the functions of these molecules is certainly warranted.

## Supplementary Information

Additional file 1: Fig. S1. Antibodies used for immunophenotyping cells in the peritoneal cavity.

Additional file 2: Fig. S2. Representative gating strategy. The figures show the representative gating strategies for myeloid (**A**) and lymphoid (**B**) cells.

Additional file 3: Fig. S3. Amino acid sequence of the recombinant Kunitz protein from* Mesocestoides corti* and two-dimensional alignment of the active loop of the Kunitz-type protease inhibitor from EgKI-1 (accession no. EUB56407.1) (Ranasinghe et al. 2018) with active site amino acids of the orthologous Kunitz-type protease inhibitor from* M. corti* (McKI-C1) (MCU_012597-RA). Blue and red letters highlight residues of the putative serine protease-binding site that comprise the conserved feature of Kunitz-type domains. Red indicates the residue in the P1 position that largely determines the specificity towards peptidase active sites. Underlined are the six cysteine residues forming disulfide bonds that stabilize the structure of the Kunitz inhibitor’s active loop. Magenta underlining indicates peptides identified by LC MS/MS analysis—the lack of Met in the N-terminal peptide likely resulted from the action of bacterial methionine aminopeptidase. Green indicates the 6×His tag (**A**). Conserved cysteine residues are highlighted in yellow, while amino acids of the active site (P6–P5’ and P18’ positions) are highlighted in blue. A tyrosine/leucine substitution in the P1 position of McKI-C1, which determines protease inhibition specificity, is indicated by a red circle (**B**).

Additional file 4: Fig. S4. SDS–PAGE gel of recombinant McKI-C1 purification steps. Lanes 1 and 2 contain two fractions after immobilized metal affinity chromatography. Lane 3 holds the Precision Plus Protein All Blue Standards (Bio-Rad). Lanes 4–6 are three fractions from the same peak after cation exchange chromatography; 8-16% pre-cast TGX gradient gel (Bio-Rad). The arrow points at the bands of recombinant McKI-C1, stained with Coomassie brilliant blue.

Additional file 5: Fig. S5. The in vitro effect of McKI-C1 on the ID8 ovarian carcinoma and B16F10 melanoma cell lines. The graphs show the effect of McKI-C1 on various attributes relevant to cancer growth and metastasis of these two cell lines, revealing no cytotoxic effect for either type of cell (**A**), and only affecting the proliferation of B16F10 (**B**). McKI-C1 did not affect the migration capabilities of ID8 or B16F10, either (**C**,** D**) and neither did it affect their ability to degrade gelatin (**E**,** F**). Comparisons of cytotoxicity and gelatin degradation were made with an unpaired* t*-test, while proliferation and wound healing were analyzed with a mixed-effects two-way ANOVA.* Ns* No statistical significance. * *p* <0.05. Cell viability:* n* = 3 independent experiments, 10 wells per cell line and condition. Wound healing:* n* = 10 wells for each cell line and condition. Proliferation:* n* = 2 independent experiments, 20 wells for each cell line and condition, imaged in four areas. Gelatin degradation:* n* = 40-50 cells scored for each cell line and condition.

Additional file 6: Fig. S6. McKI-C1-specific serum immunoglobulin levels. The graphs show the levels of serum IgM (**A**) and IgG (**B**) specific to McKI-C1, either in mice with only B16F10 melanoma (*n* = 7) (Control), those which were also inoculated with McKI-C1 (*n* = 7) (McKI-C1-treated) and those that were infected with* Mesocestoides corti* tetrathyridia for 35 days (*n* = 5) [Mc 35 days post-infection (dpi)]. The measurement of the infected mice’s serum immunoglobulins specific to whole worm homogenate was used as a positive technical control (Mc 35 dpi + McH). Cut-off values were determined from the sera of the control in accordance with Frey et al. 1998.

Additional file 7: Fig. S7. Peritoneal immune response. The graphs show the various cell populations within the peritoneal cavities of mice (*n* = 7) that were analyzed. There were no differences in the number of overall leukocytes (CD45+), B cells (CD19+), CD8+ T cells (CD8a+), CD4+ T cells (CD4+), NK cells (NK1.1+), the activation markers of CD8+ and CD4+ T cells (CD8a+ PD-1+, CD4+ PD-1+), overall myeloid cells (CD11b+), eosinophils (SiglecF+), neutrophils (Ly6G+), macrophages (CD64+), monocytes (CD11b+ Ly6C+), or the activation markers of eosinophils and macrophages (SiglecF+ PD-L1+, CD64+ PD-L1+). All of the analyses were made with an unpaired* t*-test.

Additional file 8: Supplementary Table 1. *Mesocestoides corti* transcriptome.

Additional file 9: Supplementary Table 2. KEGG pathway enrichment analysis of BL mice.

Additional file 10: Supplementary Table 3. KEGG pathway enrichment analysis of ICR mice.

Additional file 11: Supplementary Table 4. All transcripts identified as differentially expressed.

Additional file 12: Supplementary Table 5. Differential gene expression in larvae in vitro × ICR.

Additional file 13: Supplementary Table 6. Differential gene expression in larvae in vitro × BL.

Additional file 14: Supplementary Table 7. List of peptides.

Additional file 15: Supplementary Table 8. *Mesocestoides corti* proteome.

Additional file 16: Supplementary Table 9. GO term analysis.

## Data Availability

The raw data supporting the conclusions of this article will be made available by the authors, without undue reservation. The transcriptomic and proteomic data are available at https://www.ebi.ac.uk/ena/browser/home ID PRJEB97085 and http://www.ebi.ac.uk/pride ID PXD068152, respectively.

## Abbreviations

AMC: 7-Amino-4-methylcoumarin
Amp: Ampicillin
B16F10: Melanoma cell line B16F10
BL: C57BL/6J mouse tetrathyridia group
BLAST: Basic Local Alignment Search Tool
CAP: Cysteine-rich secretory proteins
DMEM: Dulbecco’s modified Eagle’s medium
EgKI-1: *Echinococcus granulosus* Kunitz inhibitor 1
ESP: Excretory-secretory products
FBS: Fetal bovine serum
GLIPR: Glioma pathogenesis-related
GO: Gene ontology
IC_50_: Half-maximal inhibitory concentration
ID8: Ovarian carcinoma cell line ID8
IgG: Immunoglobulin G
IgM: Immunoglobulin M
IV: In vitro cultivated tetrathyridia group
KEGG: Kyoto Encyclopedia of Genes and Genomes
LB: Luria-Bertani
LC/MS-MS: Shotgun liquid chromatography with tandem mass spectrometry
McES: *Mesocestoides corti* larval excretory-secretory products
McH: *Mesocestoides corti* whole larval homogenate
McKI-C1: *Mesocestoides corti* Kunitz-type chymotrypsin-specific inhibitor 1
MS: Mass spectrometry
MS–MS: Tandem mass spectrometry
NCBI: National Center for Biotechnology Information
NK: Natural killer
PBS: Phosphate-buffered saline
riMcES: Reliably identified *Mesocestoides corti* excretory-secretory products
RSEM: RNA sequencing by expectation–maximization
SDS-PAGE: Sodium dodecyl sulfate-polyacrylamide gel electrophoresis
TEAB: Triethylammonium bicarbonate
TPM: Transcripts per million
VAL: Venom allergen-like
