# Supplementary figures and images for: Integrated transcriptomic and proteomic analysis of cancer-suppressive Mesocestoides corti larvae
Source: Parasit Vectors. 2026 Apr 26;19:244. doi: 10.1186/s13071-026-07391-4 (PMC13248251; doi:10.1186/s13071-026-07391-4)

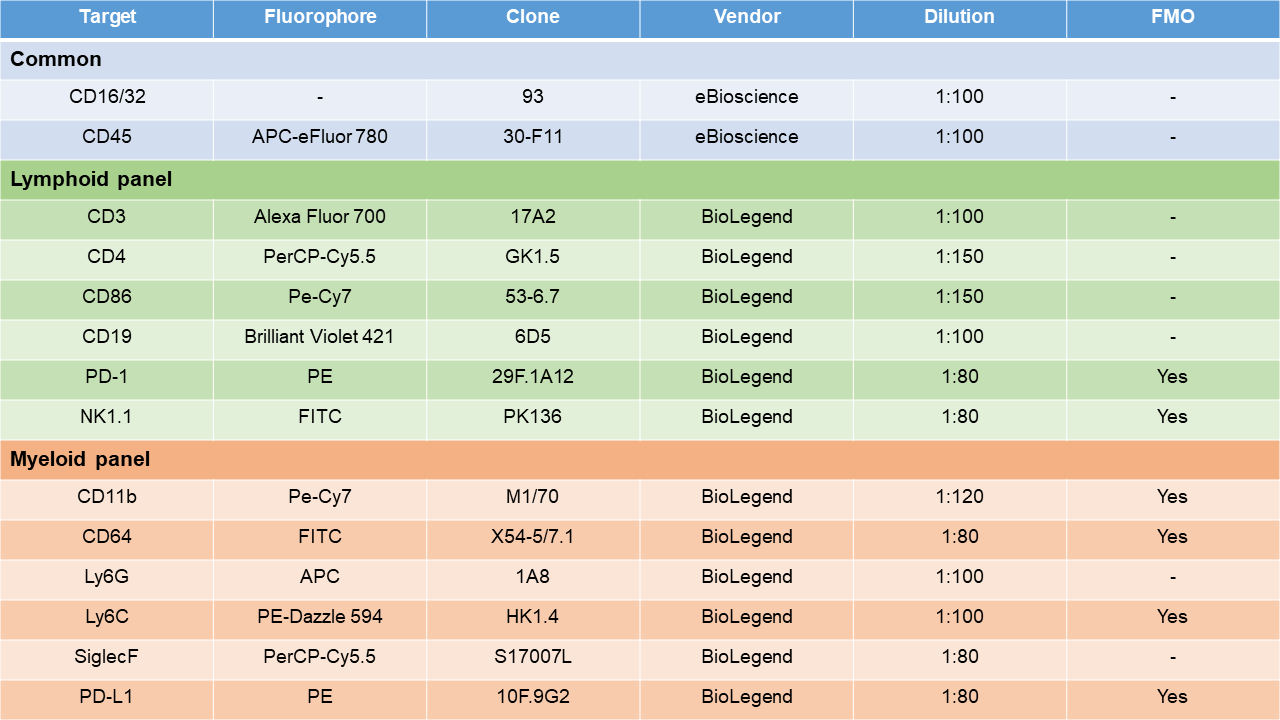

Supplement: Supplementary file 1 — Additional file 1: Fig. S1. Antibodies used for immunophenotyping cells in the peritoneal cavity. [file 13071_2026_7391_MOESM1_ESM.png]

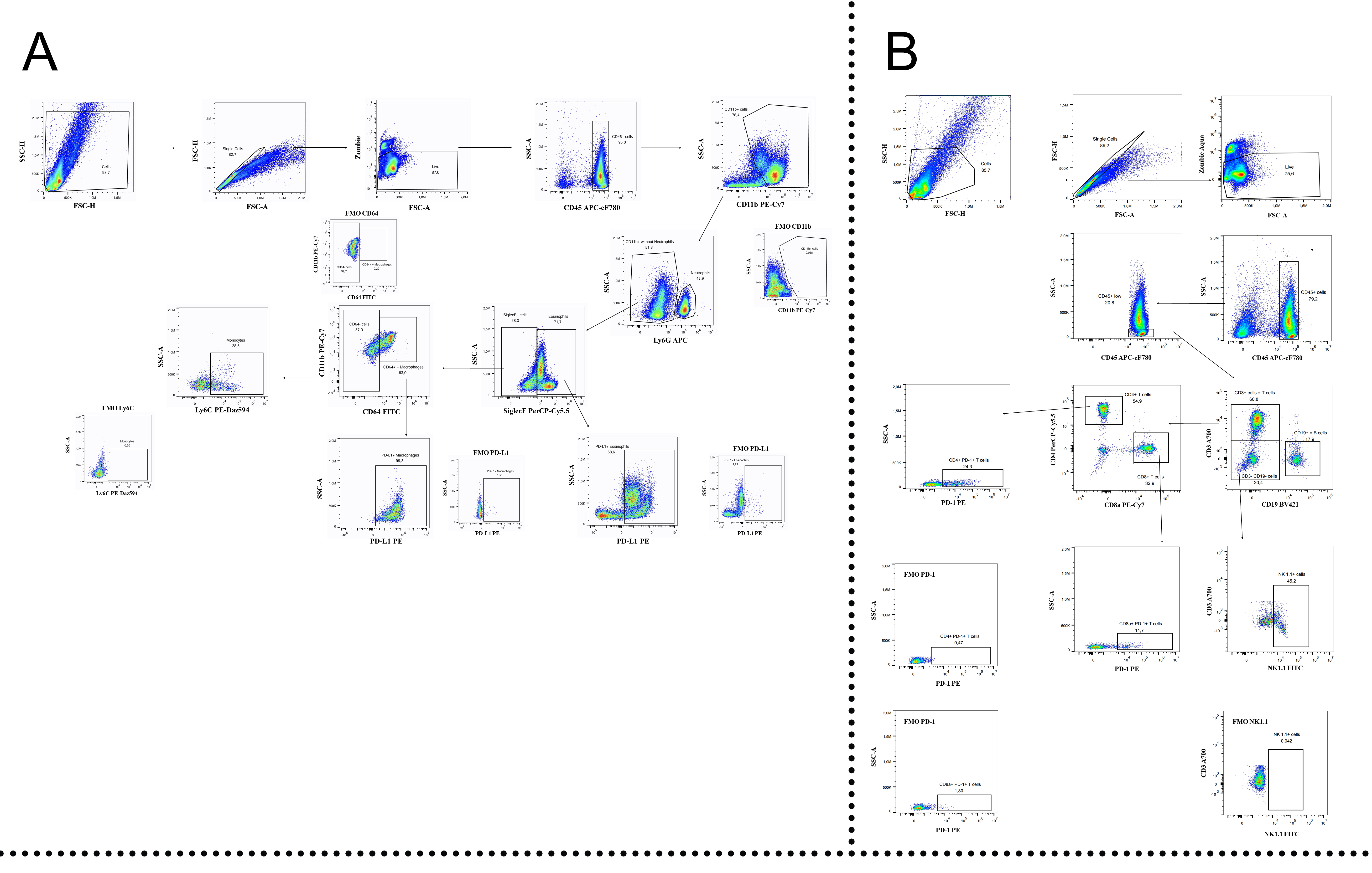

Supplement: Supplementary file 2 — Additional file 2: Fig. S2. Representative gating strategy. The figures show the representative gating strategies for myeloid (A) and lymphoid (B) cells. [file 13071_2026_7391_MOESM2_ESM.png]

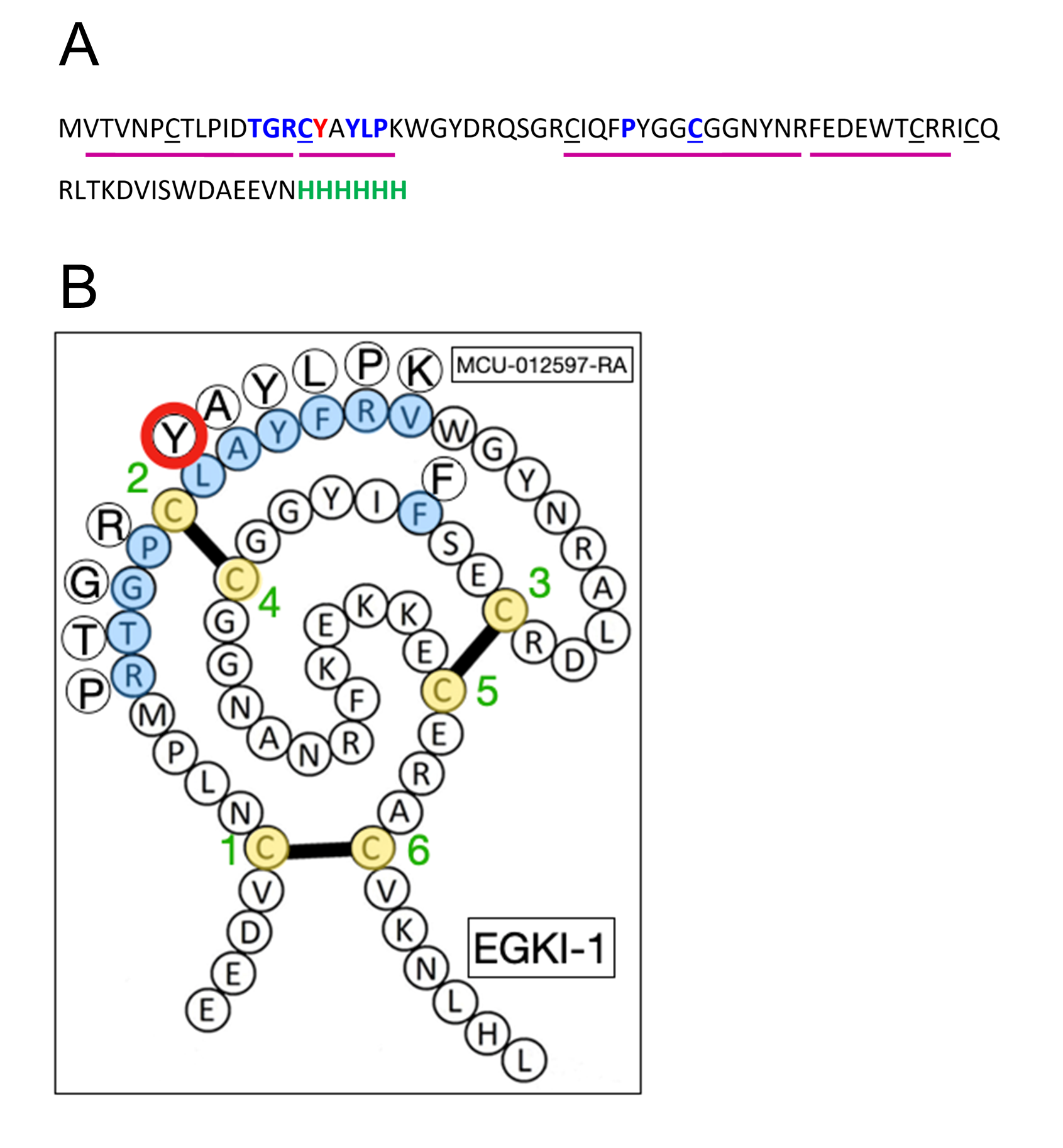

Supplement: Supplementary file 3 — Additional file 3: Fig. S3. Amino acid sequence of the recombinant Kunitz protein from Mesocestoides corti and two-dimensional alignment of the active loop of the Kunitz-type protease inhibitor from EgKI-1 (accession no. EUB56407.1) (Ranasinghe et al. 2018) with active site amino acids of the orthologous Kunitz-type protease inhibitor from M. corti (McKI-C1) (MCU_012597-RA). Blue and red letters highlight residues of the putative serine protease-binding site that comprise the conserved feature of Kunitz-type domains. Red indicates the residue in the P1 position that largely determines the specificity towards peptidase active sites. Underlined are the six cysteine residues forming disulfide bonds that stabilize the structure of the Kunitz inhibitor’s active loop. Magenta underlining indicates peptides identified by LC MS/MS analysis—the lack of Met in the N-terminal peptide likely resulted from the action of bacterial methionine aminopeptidase. Green indicates the 6×His tag (A). Conserved cysteine residues are highlighted in yellow, while amino acids of the active site (P6–P5’ and P18’ positions) are highlighted in blue. A tyrosine/leucine substitution in the P1 position of McKI-C1, which determines protease inhibition specificity, is indicated by a red circle (B). [file 13071_2026_7391_MOESM3_ESM.png]

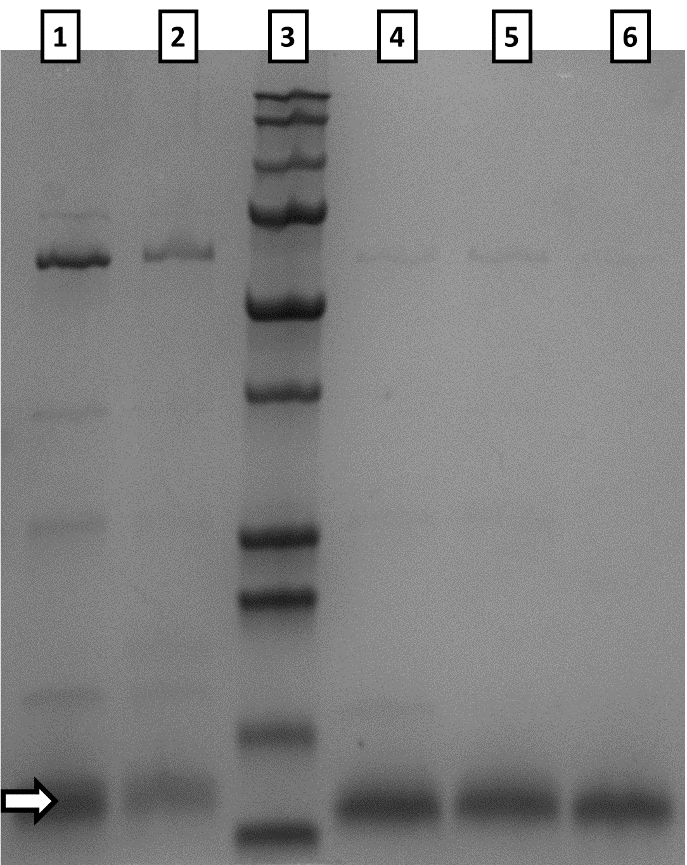

Supplement: Supplementary file 4 — Additional file 4: Fig. S4. SDS–PAGE gel of recombinant McKI-C1 purification steps. Lanes 1 and 2 contain two fractions after immobilized metal affinity chromatography. Lane 3 holds the Precision Plus Protein All Blue Standards (Bio-Rad). Lanes 4–6 are three fractions from the same peak after cation exchange chromatography; 8-16% pre-cast TGX gradient gel (Bio-Rad). The arrow points at the bands of recombinant McKI-C1, stained with Coomassie brilliant blue. [file 13071_2026_7391_MOESM4_ESM.png]

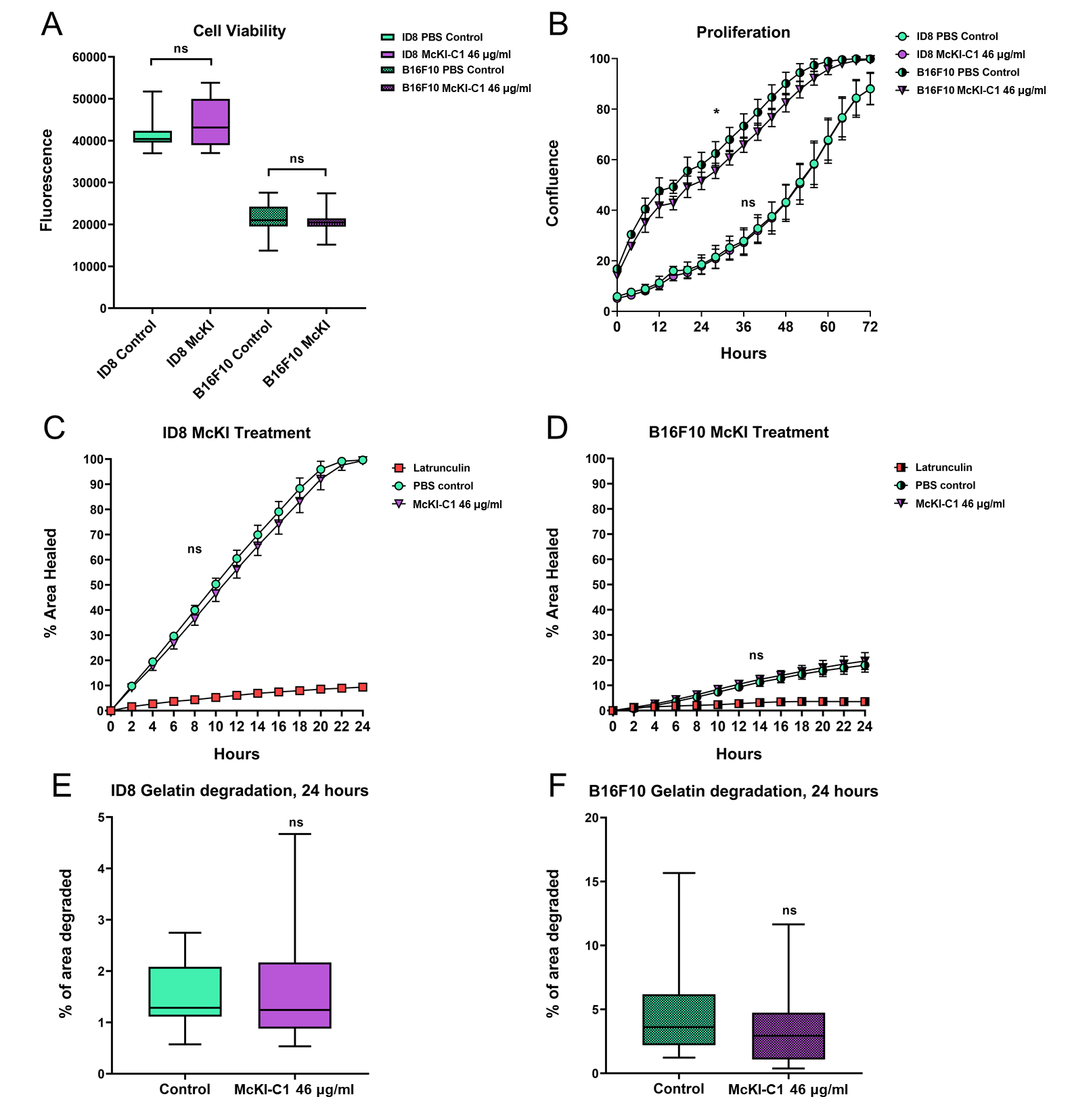

Supplement: Supplementary file 5 — Additional file 5: Fig. S5. The in vitro effect of McKI-C1 on the ID8 ovarian carcinoma and B16F10 melanoma cell lines. The graphs show the effect of McKI-C1 on various attributes relevant to cancer growth and metastasis of these two cell lines, revealing no cytotoxic effect for either type of cell (A), and only affecting the proliferation of B16F10 (B). McKI-C1 did not affect the migration capabilities of ID8 or B16F10, either (C, D) and neither did it affect their ability to degrade gelatin (E, F). Comparisons of cytotoxicity and gelatin degradation were made with an unpaired t-test, while proliferation and wound healing were analyzed with a mixed-effects two-way ANOVA. Ns No statistical significance. * p <0.05. Cell viability: n = 3 independent experiments, 10 wells per cell line and condition. Wound healing: n = 10 wells for each cell line and condition. Proliferation: n = 2 independent experiments, 20 wells for each cell line and condition, imaged in four areas. Gelatin degradation: n = 40-50 cells scored for each cell line and condition. [file 13071_2026_7391_MOESM5_ESM.png]

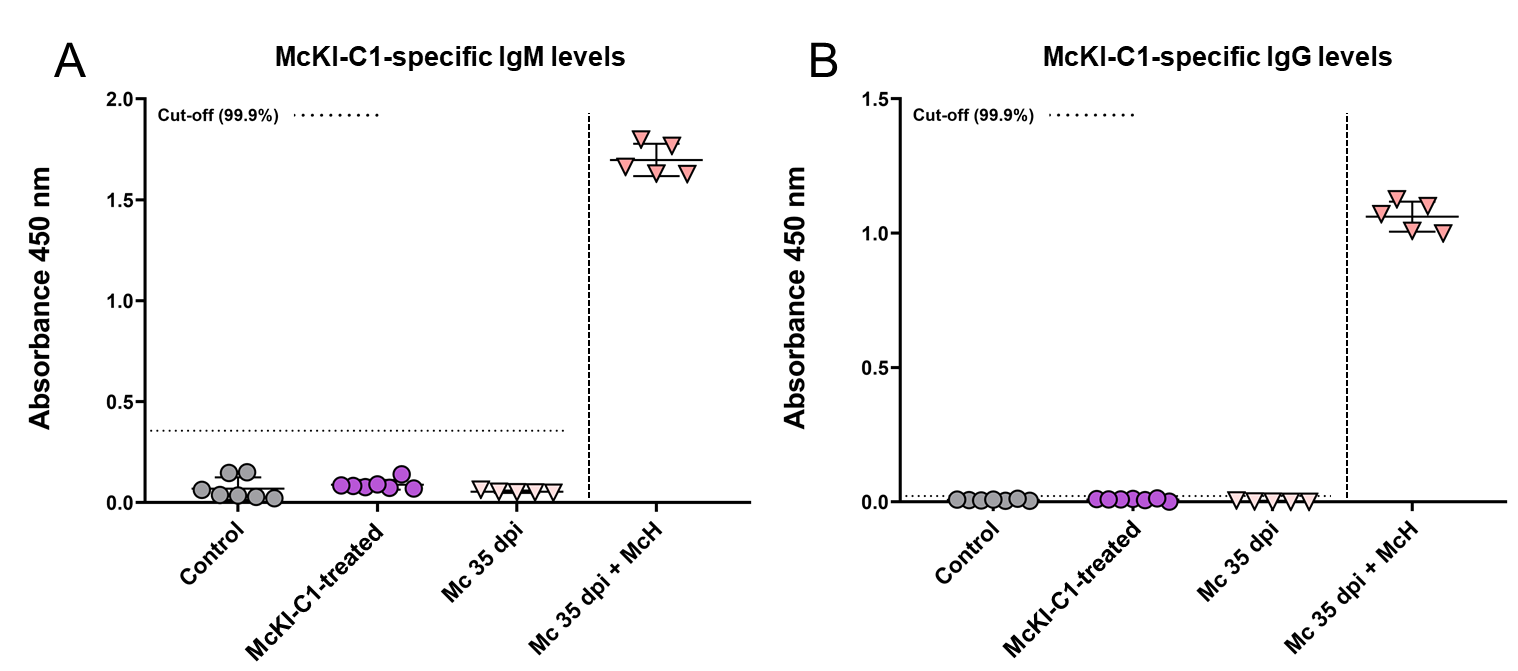

Supplement: Supplementary file 6 — Additional file 6: Fig. S6. McKI-C1-specific serum immunoglobulin levels. The graphs show the levels of serum IgM (A) and IgG (B) specific to McKI-C1, either in mice with only B16F10 melanoma (n = 7) (Control), those which were also inoculated with McKI-C1 (n = 7) (McKI-C1-treated) and those that were infected with Mesocestoides corti tetrathyridia for 35 days (n = 5) [Mc 35 days post-infection (dpi)]. The measurement of the infected mice’s serum immunoglobulins specific to whole worm homogenate was used as a positive technical control (Mc 35 dpi + McH). Cut-off values were determined from the sera of the control in accordance with Frey et al. 1998. [file 13071_2026_7391_MOESM6_ESM.png]

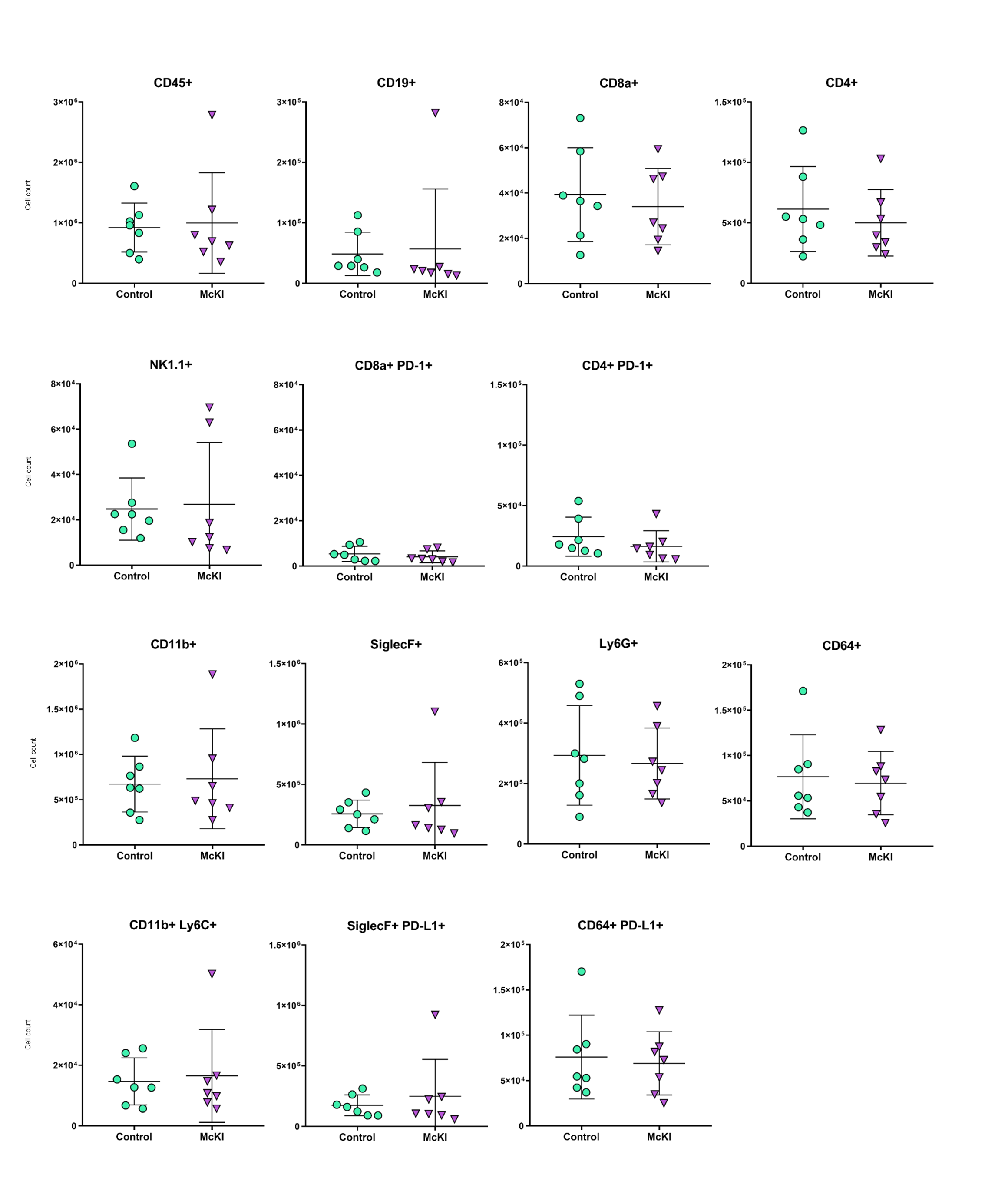

Supplement: Supplementary file 7 — Additional file 7: Fig. S7. Peritoneal immune response. The graphs show the various cell populations within the peritoneal cavities of mice (n = 7) that were analyzed. There were no differences in the number of overall leukocytes (CD45+), B cells (CD19+), CD8+ T cells (CD8a+), CD4+ T cells (CD4+), NK cells (NK1.1+), the activation markers of CD8+ and CD4+ T cells (CD8a+ PD-1+, CD4+ PD-1+), overall myeloid cells (CD11b+), eosinophils (SiglecF+), neutrophils (Ly6G+), macrophages (CD64+), monocytes (CD11b+ Ly6C+), or the activation markers of eosinophils and macrophages (SiglecF+ PD-L1+, CD64+ PD-L1+). All of the analyses were made with an unpaired t-test. [file 13071_2026_7391_MOESM7_ESM.png]
